# Dual Structural Role of Niobium in Bioactive Borate Glasses Modulates Bioactivity, Cytocompatibility, and Hemostatic Potential

**DOI:** 10.1021/acsami.5c13713

**Published:** 2025-10-24

**Authors:** Mariana Sversut Gibbin, Vitor Santaella Zanuto, Jose G. Munguia-Lopez, Robson Ferrari Muniz, Pierre Hudon, Alejandra Islas Encalada, Richard R. Chromik, Francielle Sato, Showan N. Nazhat

**Affiliations:** † Departamento de Física, 42487 Universidade Estadual de Maringá, Maringá, PR 87020-900, Brazil; ‡ Department of Mining and Materials Engineering, 5620 McGill University, Montreal, QC H3A 0C5, Canada; § Department of Bioengineering, 5620 McGill University, Montreal, QC H3A 2A7, Canada

**Keywords:** Nb, Vitreous material, Characterization, Ion release, Hemostasis, Tissue engineering

## Abstract

Designing bioactive glasses with tunable biological responses requires a precise understanding of how network modifiers influence structure–property relationships. This work investigates the effect of incorporating niobium pentoxide (Nb_2_O_5_) into melt-derived borate glasses, aiming to uncover how Nb affects the glass structure and its multifunctional biological performance. Glasses with nominal compositions: 60B_2_O_3_–(19-*x*/2)­CaO–(19-*x*/2)­Na_2_O–2P_2_O_5_–*x*Nb_2_O_5_ (*x* = 0, 2.5, 5, 7.5, and 10 wt %) were synthesized and comprehensively characterized. A key finding is the dual structural role of Nb: it predominantly acts as a network former at ≤5 wt % and as a network modifier at higher contents. This transition directly influences the glass network connectivity, as supported by physical, thermal, and vibrational techniques, and reflected a conversion from BO_4_ to BO_3_ units with an increase in Nb content, correlating with a modification in hardness and elastic modulus. Structural changes also suppressed surface reactivity and ion release, leading to a modulation in hydroxycarbonate apatite formation in simulated body fluid. On the other hand, glasses containing Nb maintained or enhanced cytocompatibility with human adipose mesenchymal stem cells. In contrast, Nb-containing glasses demonstrated reduced hemostatic potential, likely due to Nb forming niobate complexes that may influence ion exchange and clotting pathways. Overall, these findings shed light on the potential of Nb incorporation as a strategy to tailor the multifunctional properties of bioactive borate glasses for targeted biomedical applications and offer a fresh perspective on how the dual structural role of niobium contributes to their overall performance within the context of biomaterials.

## Graphical abstract

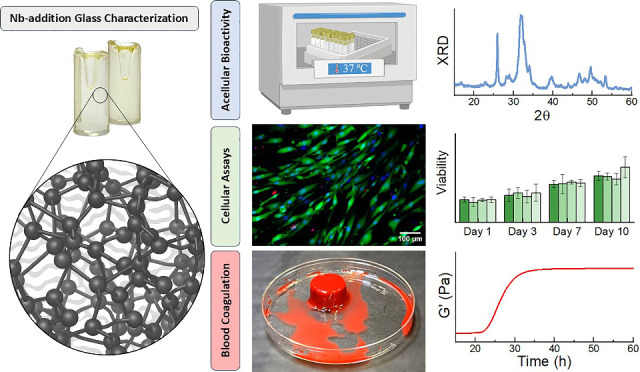

## 1 Introduction

Bioactive glasses represent a class of biomaterials with great potential for applications in hard and soft tissue regeneration due to their ability to interact with host tissues.^1–3^ Since the introduction of Bioglass^Ⓡ^ 45S5 in the 1970s, various compositions have been developed based on different glass systems, such as silicates, phosphates, borates, and borosilicates, among others, aiming to optimize their physicochemical properties.^2,3^ Each of these systems confers unique structural and compositional features, directly influencing their solubility, ionic release profiles, and cellular responses. This versatility stems from the compositional and structural flexibility of glasses, enabling precise tuning of their properties by adjusting the chemical composition and network structure.^3,4^

Borate-based glasses have been attracting increasing interest due to their high degradation rate in biological environments, promoting the rapid and controlled release of bioactive ions.^5,6^ These characteristics are particularly advantageous for applications that require accelerated cellular responses, such as soft tissue regeneration, angiogenesis, wound healing and even as hemostatic agents.^7,8^ However, the high solubility of these glasses may compromise their structural stability and limit their applications.^7^ To overcome these limitations, various network modifiers have been incorporated into the glass structure to tailor their properties without compromising their bioactivity.^4,7,9^

Magnesium,^10^ zinc,^11^ copper,^11^ strontium,^10^ titanium,^12^ among others, have been successfully used as modifiers. The introduction of these elements can directly influence the bond density within the glass network, thereby altering material properties, such as dissolution rates, biocompatibility, cellular responses,^10–12^ and antimicrobial activity, as in the case of silver.^13,14^ The selection and content of these glass modifiers play a critical role in determining glass functionality and suitability for specific applications.^4,15^

Niobium (Nb) has been explored as an additive in silicate glasses with promising results.^1,4,15–18^ However, Nb acts not only as a network modifier but also as a network former, depending on its concentration and the host matrix, potentially performing both roles simultaneously. This dual behavior brings several advantages to silicate-based matrices.^15,19^ In this regard, the incorporation of niobium oxides (such as Nb_2_O_5_) into silicate glasses has demonstrated several benefits, including improved microhardness,^20,21^ modulation of degradation rates,^2,4,17^ enhanced apatite formation,^15,18^ cytocompatibility,^1,4,9,17,18^ angiogenic response,^1,15^ as well as osteoconductive and osteoinductive activities.^4,9,15,17,18^ These properties make Nb a potentially attractive candidate for incorporation into bioactive glasses.

Although Nb has been previously investigated in borate glass systems, mainly for its structural and optical contributions, its effect and impact on bioactivity, cytocompatibility, and hemostatic properties remain largely unexplored. Specifically, no studies have systematically examined the effect of Nb incorporation into bioactive borate glasses with respect to biological properties, and, in particular, on how the dual role of Nb influences these properties, especially given the various conformations adopted by boron units. In light of the unique structural characteristics of borate glasses and their high biological potential,^5,22^ the incorporation of Nb_2_O_5_ may represent a promising strategy for developing bioactive glasses with enhanced properties for various biomedical applications. Therefore, the present study reports on the synthesis and characterization of bioactive borate glasses with varying Nb_2_O_5_ content, aiming to assess their structural and physicochemical properties as well as biological impact.

## 2 Materials and Methods

### 2.1 Sample Preparation

Nb-incorporated borate glasses were synthesized using high-purity grade oxides (B_2_O_3_ and P_2_O_5_ with 99.98% from Sigma-Aldrich; CaCO_3_ with 99.95% and Na_2_CO_3_ with 99%, both from Alfa Aesar; and Nb_2_O_3_ with 99%, which was provided by *Companhia Brasileira de Metallurgia e Mineração,* Brazil). Na_2_CO_3_ and CaCO_3_ were degassed at 450 and 900 °C for 2 h to obtain Na_2_O and CaO, respectively. Glasses were synthesized using the melt-quenching technique in nominal compositions of 60B_2_O_3_–(19-*x*/2)­CaO–(19-*x*/2)­Na_2_O–2P_2_O_5_–*x*Nb_2_O_5_ (*x* = 0, 2.5, 5, 7.5 and 10), in wt %, as detailed in Table 1. The effective glass compositions estimated by X-ray fluorescence spectroscopy (XRF) are provided in Table S1 (Supporting Information).

**Table 1:** Glass Sample ID and Nominal Compositions (wt % and mol %)

	B_*2*_O_3_		CaO		Na_2_O		P_2_O_5_		Nb_2_O_5_	
Sample	wt %	mol %	wt %	mol %	wt %	mol %	wt %	mol %	wt %	mol %
PNCB	60.00	56.65	19.00	22.27	19.00	20.15	2.00	0.93	0.00	0.00
Nb-PNCB:1	60.00	57.91	17.75	21.27	17.75	19.24	2.00	0.95	2.50	0.63
Nb-PNCB:2	60.00	59.22	16.50	20.21	16.50	18.29	2.00	0.97	5.00	1.29
Nb-PNCB:3	60.00	60.60	15.25	19.12	15.25	17.30	2.00	0.99	7.50	1.98
Nb-PNCB:4	60.00	62.04	14.00	17.97	14.00	16.26	2.00	1.01	10.0	2.70

The batches were thoroughly mixed and homogenized using an agate mortar and then melted in a single-step process at a target temperature of 1200 °C for 30 min in a platinum crucible, under air furnace conditions. A relatively high heating rate of 20 °C/min and a short holding time at eutectic temperature were applied to minimize volatilization and compositional losses during melting. The melts were then immediately poured into a 10 mm diameter stainless-steel mold and preheated to 470 °C for 2 h. The resulting glass products underwent annealing in a muffle at the same temperature for 6 h to release the internal stresses generated through the quenching process. Glass monoliths were either sectioned into 2 mm thick discs, which were polished with SiC abrasive paper, using isopropyl alcohol, for radiopacity and nanoindentation analyses, or ground using an agate mortar and pestle and sieved to a particle size range between 25 and 75 μm for further analysis.

### 2.2 Structural and Physical Characterization

The radiopacity of the glass discs samples were characterized through digital radiography using a X70 dental X-ray device (Xdent) at 70 kV, 8 mA, and 90 ms exposure time. Radiographic images were acquired on glass samples fixed over a New Ida Dabi Atlante digital sensor (Eagle) 20 × 30 mm at a distance of 15 cm from the device, with the area shielded with lead to ensure radiological protection during X-ray exposure. Radiopacity assessment was performed by analyzing the grayscale levels of the images compared with an aluminum penetrometer. The histogram tool in Adobe Photoshop CS6 software was used to quantify the average gray intensity values of Al penetrometer, on a scale from 0 (black) to 255 (white), and was used to estimate the radiopacity in mmAl for each sample.

Particle size fractions (*D*_50_) of the glass powders were determined by using a Microtrac SYNC particle size detector (ATS Scientific Inc.). Textural properties were measured with nitrogen gas adsorption and desorption isotherms collected using a TriStar II Plus Automatic Physisorption Analyzer (Micromeritics Instrument Corporation) gas sorption system (*n* = 3). Specific surface area (SSA) values were determined using the Brunauer–Emmett–Teller (BET) method,^23^ while the average pore volume (PV) values were calculated using the Barrett–Joyner–Halenda (BJH) method.^24^

XRF analysis was performed on as-made, pristine materials and powdered glasses using an Epsilon 1 (Malvern Panalytical) with a silver anode (Ag) in the Ominian mode. Compositions of each oxide was determined using equipment software.

Glass density (ρ) was measured on discs using the Archimedes method in distilled water at room temperature, via the equation: ρ = *ρ*_water_·(*W*_air_/(*W*_air_ – *W*_water_)), where *W*_air_ and *W*_water_ are the density of distilled water at room temperature.

Differential scanning calorimetry (DSC) was performed on 40 mg powder samples placed in a platinum crucible and heated from room temperature to 1100 °C, at 10 °C/min under a N_2_ atmosphere with a flow rate of 20 mL/min using a STA 449 F3 Jupiter thermal analysis apparatus (Netzsch).

X-ray diffraction (XRD) patterns were obtained in glass powders with a D2 Phaser diffractometer (Bruker) using CuKα radiation (λ=1.5418 Å) in 2θ configuration, an angular step of 0.02° and a time of 2 s/step in the angular range of 10–60°, operating with a voltage of 40 kV and a current of 30 mA.

Attenuated total reflectance-Fourier transform infrared (ATR-FTIR) spectroscopy of the glass particles was carried out between 4000 and 400 cm^–1^ with a resolution of 4 cm^–1^ and 128 scans using a Vertex 70 V spectrometer with ATR diamond crystal plate (Bruker Optik GmbH & co.). The spectra were baseline corrected and normalized by the normalization vector using the OPUS software.

Raman spectra were obtained using a Senterra Confocal Raman microscope (Bruker Optik GmbH & co.) equipped with a 532 and 785 nm laser excitation source. The laser power was 20 and 100 mW, respectively, focused on the sample with a 20× magnification lens to analyze the glasses before and after SBF immersion, respectively. A spectral resolution of 3 to 5 cm^–1^ was used in the range of 1750 to 400 cm^–1^, using 30 spectra/sample with a detector integration time of 10 s, normalized by the normalization vector using the OPUS software.

Hardness (H) and Elastic Modulus (E) values were generated using nanoindentation tests performed with an NHT2 instrument (Anton Paar), equipped with Berkovich indenter (ϵ = 0.74). A Poisson’s ratio (ν) of 0.29 (as reported in^25^) was used for all calculations. Load and displacement were continuously recorded during the indentation process, generating loading–unloading curves, and used to determine H and E by means of the Oliver and Pharr procedure.^26^ Each glass specimen (*n* = 2) was carried out in a series of 60 indentations, interspersing 15 points for each load (10, 50, 100, and 200 mN) and arranged in a 5 × 12 matrix, based on D’Andrea et al.^27^ The acquisition rate for the nanoindentation was 10 Hz, applying the respective load at a rate of 150 mN/min, kept for 3 s, and discharged up to 10% by a period of 10 s.

### 2.3 Aqueous Interactions, Ion Release, and Acellular Bioactivity in Simulated Body Fluid

Aqueous interactions of the glass particles were investigated through dynamic vapor sorption (DVS) using a DVS Resolution (Surface Measurement Systems Ltd.), which measures mass changes (±0.1 μg) under controlled relative humidity (RH) and temperature. Glass particles (10 mg) were placed in an aluminum pan and inserted into a chamber at 25 ± 0.05 °C and directly exposed to 90% RH for 24 h, followed by 0% RH for a further 24 h to obtain the sorption and desorption curves, respectively.

The release of boron, calcium, sodium, phosphorus and niobium ions from glass particles (*n* = 3) were measured in deionized water (DIW) at a 1.5 mg/mL ratio^28^ using an iCAP 6500 inductively coupled plasma–optical emission spectrophotometer (ICP-OES; Thermo Scientific). DIW was filtered through a 0.2 μm nylon filter after 1 h, 6 h, 1 day, and 3 days of immersion and then stored in a 15 mL falcon tube followed by 1:1 dilution with 4% (w/v) nitric acid (Fisher Scientific). Serially diluted solutions of boron (0, 0.5, 5, 50 ppm), calcium (0, 0.5, 5, 50 ppm), sodium (0, 0.5, 5, 50 ppm), phosphorus (0, 0.5, 5, 50 ppm), and niobium (0, 0.1, 1, 10 ppm) were used as standards (Fisher Scientific). Change in solution pH due to glass dissolution was measured (*n* = 3) at the same time points with an Orion Star A211 pH meter (Thermo Scientific).

Kokubo’s simulated body fluid (SBF) was used to examine the mineralization capability of the glasses.^29^ Glass particles were added to sterile 50 mL falcon tubes containing SBF (pH 7.4; replaced every 2 days) at a ratio of 1.5 mg/mL and stored in a KSI 4000 I Control incubator shaker (IKA) at 120 rpm and 37 ± 1 °C for 1 h, 6 h, 1 d, 7 d, and 14 d (*n* = 3).^30^ The remaining glass was rinsed twice with DIW and anhydrous ethanol before drying in an oven at 60 °C for 1 day and subjected to XRD, ATR-FTIR and Raman spectroscopy as well as Scanning electron microscopy (SEM) and energy-dispersive X-ray Spectroscopy (EDS) analyses. SEM-EDS characterizations were performed by a scanning electron microscopy model FEI Scios DualBeam (Thermo Scientific). Before SEM-EDS, the samples were coated with gold using a sputter coater model SCD 050 (Bal-Tec).

### 2.4 Cellular and Biocompatibility Assays

Human adipose-derived mesenchymal stem cells (huAD-MSCs, ATCC, PCS-500-011) were cultured in Dulbecco’s modified eagle’s medium (Gibco) supplemented with 1% penicillin/streptomycin (Gibco) and 10% (v/v) fetal bovine serum (HyClone) and incubated at 37 °C under 5% CO_2_. Cells were expanded once they reached 70% confluency using Trypsin-EDTA solution (0.05%; Gibco). Tests were performed on cells between passages 4 and 6.

Cells were seeded at a density of 4000 cells/well in 96-well plates and incubated for 24 h before being exposed to growth media enriched with ionic dissolution products of PNCB, Nb-PNCB:1 and Nb-PNCB:4, which were generated after 6 h of dissolution in supplemented medium, at 1.5 mg/mL, and filtered through 0.2 μm nylon filter. At days 1, 3, 7 and 10, cultures were washed three times with Dulbeccos’s phosphate-buffered saline (D-PBS; Gibco) and cell viability and metabolic activity were determined using Live/Dead and WST-1 assays, respectively, as per Lepry et al.^31^

The Live/Dead assay was performed using a solution of 1 μL of calcein-AM (4 mM, AAT Bioquest) and 2 μL of EthD-1 (2 mM, Tocris Bioscience) diluted in 1 mL of D-PBS. Hoechst 33342 (18 mM, Tocris Bioscience) was added to the solution as a nuclear stain (2 μL). After incubation at 37 °C for 30 min, microscopic images were generated using an Axio Observer 5 fluorescence inverted microscope (Carl Zeiss). All conditions were tested in triplicate.

Cell metabolic activity was assessed using the cell proliferation reagent WST-1 (Roche, Sigma-Aldrich) according to manufacturer instructions. Each well received 100 μL of fresh culture medium and 10 μL of WST-1 reagent, followed by incubation at 37 °C for 2 h, before its absorbance was measured at 440 nm using a microplate reader.

### 2.5 Blood Coagulation

The ElastoSens^TM^ Bio^2^ instrument (Rheolution Inc.) was employed to characterize the hemostatic potential of glass particles and their ionic dissolution products by measuring changes in the shear storage modulus (*G*′) of blood in real time.^32,33^ Each 6 mL sample consisted of 4 mL of CL1700–500C citrated whole bovine blood (Cedarlane) and: 1) 2 mL of DIW containing either 7 or 14 mM of glass (PNCB and Nb-PNCB:4) dissolution products (as determined by ICP); 2) glass particles at either 15 or 30 mg/mL, which were directly added to the sample holder containing 2 mL of DIW and 4 mL of citrated blood. Calcium chloride (CaCl_2_, Sigma-Aldrich) in 7 and 14 mM were used as positive controls, whereas the coagulation behavior of citrated whole bovine blood without CaCl_2_ was monitored as a negative control. No significant interference from the sample holder with the coagulation process was observed.

Tests were standardized based on the protocols described by Rezabeigi et al.^33^ and Naseri et al.^34^ and conducted in triplicate. Prior to testing, the blood was incubated at 37 °C for at least 30 min. The bovine blood used was sourced from a single 500 mL batch, stored at 4 °C, and utilized within a maximum period of 7 days. *G*′ values were recorded over 60 min with temporal resolution steps of 30 s. Temporal changes in *G*′ were used to determine the activated coagulation time, which was defined as the point at which *G*′ increased sharply.

### 2.6 Statistical Analysis

Statistical analysis was conducted using Prism 7 (Graphpad). The mechanical tests and cellular assay data followed a normal distribution (Shapiro-Wilk test), and a two-way ANOVA analysis was performed, followed by Tukey’s test (*p* < 0.05). All figure error bars indicate standard deviations.

## 3 Results and Discussion

### 3.1 Textural and Physical Characterization

Figure S1 (Supporting Information) shows photographic images of the glass monoliths which suggested that all samples were macroscopically homogeneous. In contrast, digital radiography of the glasses displayed distinct color patterns that progressively changed with an increase in Nb content (Figure S2 and Table 2). The presence of Nb directly influences X-ray absorption, which can be associated with its intrinsic density and atomic mass.^35^ As a transition metal of relatively high atomic number and density, Nb incorporation leads to an increase in density,^15,36^ and enhances its ability to attenuate X-ray radiation. This increase in radiopacity is evident in the lighter regions observed in samples of higher Nb content, which tend to be more opaque to X-rays, whereas those with lower Nb content show greater transparency.

**Table 2:** Characterization of the As-Made Glasses^a^

Sample	PNCB	Nb-PNCB:1	Nb-PNCB:2	Nb-PNCB:3	Nb-PNCB:4
Radiopacity (mmAl)	1.38	2.18	2.75	3.48	4.77
*D*_50_ (μm)	64.7	65.1	68.1	65.4	63.7
SSA (m^2^/g)	0.10	0.11	0.10	0.09	0.11
Pore width (nm)	2.20	6.38	5.57	5.55	5.31
Pore volume (10^–4^ cm^3^/g)	5.84	5.83	5.03	5.51	6.04
ρ (g/cm^3^) ± 0.01	2.52	2.56	2.57	2.57	2.57
APF	0.599	0.606	0.610	0.609	0.608
*T*_g_ (°C)	510	513	523	522	520
*T*_x_ (°C)	635	662	663	680	711
Δ*T* (*T*_x_ – *T*_g_)	125	149	140	158	191

a Average diameter (*D*_50_), specific surface area (SSA), average pore width and average pore volume, density (ρ), atomic packing factor (APF), and thermal properties (glass transition temperature (*T*_g_), onset of crystallization temperature (*T*_x_) and thermal stability parameter (Δ*T*)).

This trend has been widely reported in literature on restorative and glassy biomaterials, where the presence of elements with higher atomic numbers is directly associated with increased radiopacity, an essential parameter for clinical applications, particularly in areas requiring radiographic monitoring and clear distinction of the material from adjacent anatomical structures, such as in dentistry.^21,35,37,38^ In Nb-PNCB:3 and Nb-PNCB:4, lighter regions were identified in the radiographic images, indicating a higher local absorption of radiation. Therefore, although the samples appear as visually homogeneous, there is evidence that the addition of Nb may be influencing the viscosity of the eutectic during melting and promoting the formation of clusters or microdomains with higher concentrations of metal oxides.^39^

All compositions were ground to a similar median particle size (*D*_50_) of 65 ± 1 μm to directly compare their textural properties (Table 2). SSA values was approximately 0.10 m^2^/g for all samples, which is within the expected range for melt-quenched glasses,^5^ and substantially lower than those generated through chemical routes.^5^ The pore width increased from 2.20 to 6.38 nm when comparing PNCB and Nb-PNCB:1 but showed a slight reduction with a further increase in the Nb content, decreasing to 5.31 nm in Nb-PNCB:4. This behavior may indicate a progressive reorganization of the glass network, possibly due to the dual role of Nb as a network modifier, which can lead to increased cross-linking or the formation of Nb-rich microdomains that limit further pore expansion.^19^ Conversely, the total pore volume remained relatively constant across all compositions, averaging at 5.7 ± 0.4 cm^3^/g. These observations suggest that while Nb content influences pore size distribution, it has a minimal impact on overall pore volume under the used processing conditions.

Glass density (ρ) gradually increased with increasing Nb content, reaching a plateau tendency above Nb-PNCB:2 (Figure 1a). Densification rate increased by almost 2% compared to PNCB, which may be attributed to the higher density (4.6 g/cm^3^) and molecular weight (265.81 g/mol) of Nb_2_O_5_ compared to the other glass components. Additionally, the replacement of CaO and Na_2_O by Nb_2_O_5_ contributes to this densification effect.^15,36^ Above Nb-PNCB:2_,_ the rate of density increase becomes negligible, indicating a potential saturation point in the structural accommodation of Nb within the glass network.^19^ At higher content, Nb may shift from a predominantly network forming to a modifying role.^19^ This transition can result in an increased formation of non-bridging oxygens (NBOs) and localized structural distortions, which reduce the packing efficiency of the network. Consequently, the competing effects between the mass increase and structural expansion lead to a stabilization of the glass density. This trend reflects a limit in the densification capacity of the glass matrix upon continued addition of Nb_2_O_5_, as governed by structural reorganization mechanisms.

**Figure 1:**
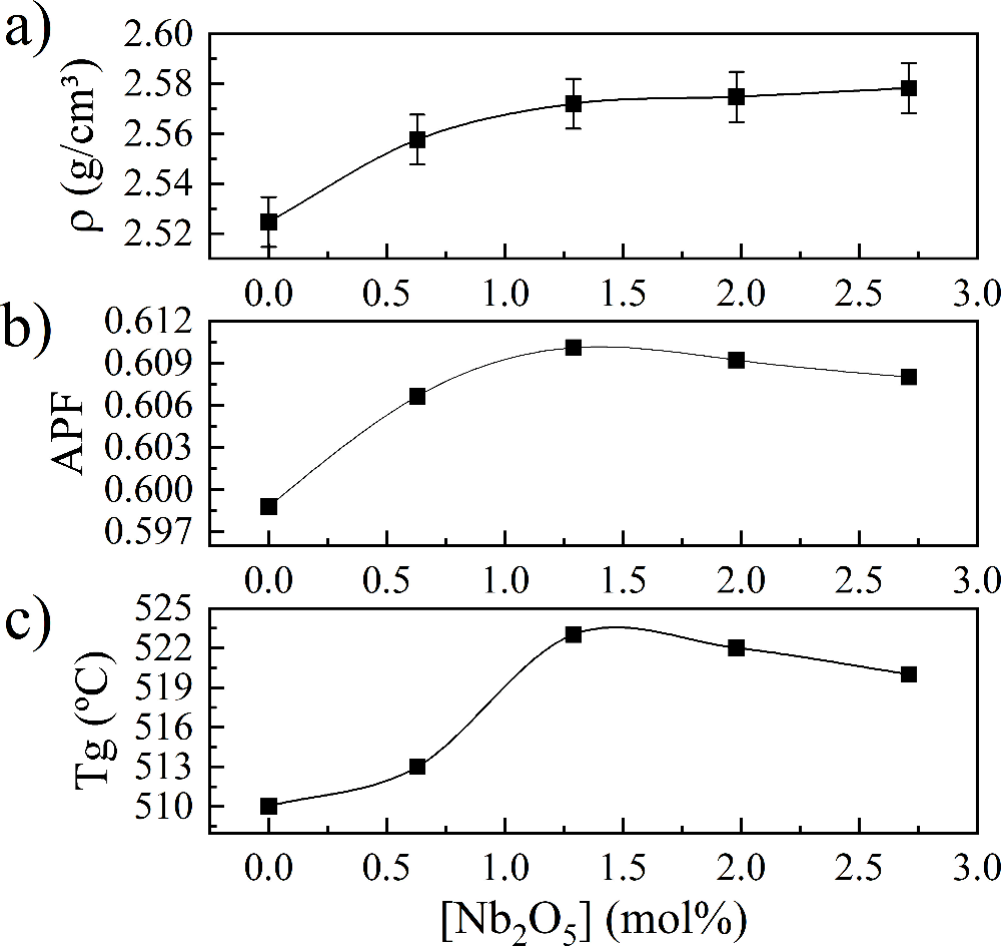
a) Density, b) atomic packing factor (APF), and c) glass transition temperature (*T*_g_) of glasses as a function of Nb_2_O_5_ content.

The atomic packing factor (APF) of the glasses was calculated according to Smedskjaer et al.^40^ as an indicator of the efficiency of their atomic arrangement. Although amorphous, APF may be used to describe the average atomic density of the glasses. In particular, the introduction of different oxides, such as Nb_2_O_5_ in borate glasses, can modify the APF by altering the glass network atomic coordination and spatial occupancy, impacting its physical and mechanical properties.^40^

It was found that the addition of Nb_2_O_5_ at the expense of CaO and Na_2_O resulted in an increase in the APF from 0.599 to 0.610 in Nb-PNCB:2 (Figure 1b). This suggests that a larger fraction of the glass total volume is occupied by atoms, resulting in a denser structure. This behavior indicates that Nb is increasingly participating in the glass network as a network-forming component, contributing to a more compact atomic arrangement.

On the other hand, the decrease in APF from 0.610 in Nb-PNCB:2 to 0.608 in Nb-PNCB:4 suggests an anomalous trend. This reduction in the APF indicates that excess Nb incorporation promotes the formation of structural defects, causing a shift in the role of Nb from a network former to a network modifier, disrupting the glass network and thereby compromising structural compactness. A similar behavior has been observed in soda-lime-borate systems, which reached an APF of 0.597 followed by a decrease to 0.595 between compositions of 15 and 35 mol % Na_2_O.^40^ It is worth noting that the density of these glasses also plateaued at a higher Na_2_O content at the expense of the network-forming B_2_O_3_. Initially, Na_2_O contributed to the densification of the network by converting BO_3_ into more efficiently packed BO_4_ units. However, beyond a critical concentration (∼25 mol %), the formation of NBOs dominates, leading to a decrease in network connectivity and packing efficiency. Moreover, it was shown that the change in APF significantly affected glass thermal and structural properties.^40^

### 3.2 Thermal Characterization

Figure S3a (Supporting Information) shows the DSC curves of the glasses with the main transition temperatures summarized in Table 2. The single *T*_g_ confirmed the homogeneity of the as-made glasses, which is reported in Figure 1c as a function of Nb_2_O_5_ content, revealing a clear compositional dependence and reflecting changes in the network.

Glasses with lower Nb_2_O_5_ contents (*i.e.*, up to Nb-PNCB:2), demonstrated an increase in *T*_g_, which can be attributed to Nb incorporation as a network former. In this region, Nb predominantly exists as NbO_6_ octahedra that interconnect directly with the network via covalent linkages. This leads to an increase in the cross-linking density of the glass network due to the shared corners of the octahedra. Given the high bond strength of Nb–O bonds, the presence of these units raises the energetic barrier for structural relaxation, thereby increasing in *T*_g_.^15^

At higher Nb_2_O_5_ contents, there may be an excess of NbO_6_ units that must be accommodated in the structure, leading to a localized structural distortions, increasing the formation of NBOs.^15,19^ Such changes signal a partial transition of Nb from a network former to a network modifier, disrupting the continuous network and reducing the overall packing efficiency, as also observed in the APF values of Nb-PNCB:3 and Nb-PNCB:4. As a result, *T*_g_ shows a slight decrease, reflecting a more depolymerized structure.

Furthermore, the presence of Nb-rich structural motifs within the glass matrix may hinder the nucleation of crystalline phases, contributing to the observed increase in *T*_x_ and in the thermal stability parameter (ΔT) (Table 2). The continued increase in *T*_x_, even as *T*_g_ slightly decreases, suggests that these Nb-incorporated structures disrupt the network in a manner that impedes crystallization. This behavior is similar to that observed in glasses containing network modifiers, where structural disruption does not necessarily lead to crystallization, due to the decoupling between network relaxation and the diffusion kinetics of the modifier ions.^40^ Overall, the thermal properties also reflect the dual role of Nb, which tends to act as a former at lower contents, transitioning towards a modifier role at higher contents and influencing both *T*_g_ and *T*_x_ through structural depolymerization.

### 3.3 Structural Characterization

XRD analysis was carried out on the as-made glasses (Figure S3b; Supporting Information) with the diffractograms revealing no detectable peaks and displaying a broad diffuse scattering that confirms their amorphous nature.^41,42^

ATR-FTIR spectroscopy was used to analyze the molecular structure of the glasses (Figure 2a). It was possible to identify the three main regions that are associated with borate-based glasses. In particular, the peaks between 1200 and 1500 cm^–1^ can be attributed to the vibrational mode of B–O stretching in BO_3_, while the B–O stretching of BO_4_ units are present at 850–1200 cm^–1^ and B–O–B bending of BO_3_ at ∼700 cm^–1^.^43–45^ The broad band at approximately 950 cm^–1^ signifies the B–O linkages within BO_4_, whereas the B–O stretching of boroxol rings is highlighted by the shoulder around 870 cm^–1^.^46–49^

**Figure 2:**
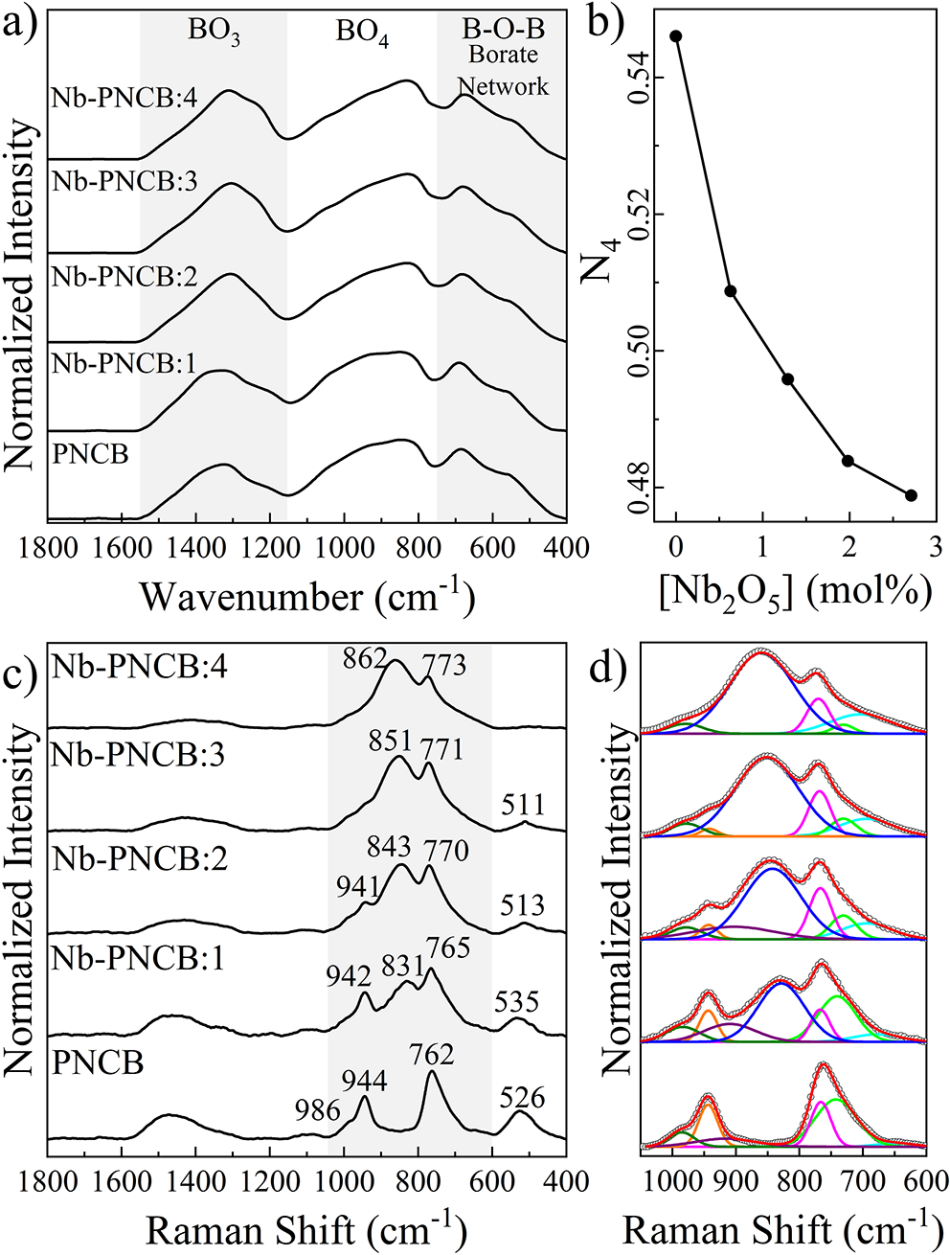
Structural characterization of Nb-glasses. a) ATR-FTIR spectra, b) relative peak areas (Ar) for the 3- and 4-coordinated bonding regions (line is used as a guide), c) Raman spectra, and d) Gaussian deconvolution of the Raman spectra to determine structural changes through Nb addition.

It is possible to use the peak integration related to BO_3_ and BO_4_ to provide semiquantitative analysis associated with boron coordination. The N_4_ borate basic units are believed to reflect the network connectivity of the borate-based glasses. Although the N_4_ fraction is generally determined by boron nuclear magnetic resonance spectroscopy, FTIR spectroscopy can be used to estimate this through the equation: N_4_ = *A*_r_/(α + *A*_r_), where *A*_r_ is the ratio between the areas associated with boron tetrahedra and boron triangles and α is the relative integrated absorption coefficient of 4-coordinated versus 3-coordinated boron. However, this coefficient may change depending on composition.^42^ For example, Hübert et al.^50^ reported α = 1.3 for SrO-B_2_O_3_,^50^ whereas Lepry and Nazhat obtained α = 1.5 for sol–gel calcium-borate systems^22^ and Yiannopoulos et al.^51^ reported α = 1.9 for glasses containing Mg. Therefore, it is possible to consider an intermediate value for the calculation as a slight approximation, as shown in Figure 2b.

A progressive reduction in BO_4_ units was observed with increasing Nb_2_O_5_ content. This indicates depolymerization of the borate network, likely due to a partial conversion of BO_4_ to BO_3_ units. Nevertheless, the concurrent increase in glass density and APF up to Nb-PNCB:2, together with the emergence of vibrational bands assigned to NbO_6_ (as also discussed via Raman spectroscopy below), suggests that Nb^5+^ initially incorporates into the network as a conditional former. In this role, NbO_6_ contributes to network densification and structural reinforcement due to their larger coordination volume, resulting in greater connectivity of the glass network, even as BO_4_ units decrease. At higher Nb_2_O_5_ contents, the continued reduction in BO_4_, decrease in APF and *T*_g_ as well as plateauing of density all reflect a transition of Nb to a network modifier role.

The modification in glasses by Nb addition can also be evidenced by the Raman spectra, which exhibited changes in vibrational modes, another indication of the incorporation of Nb into the glass network (Figure 2c).

It is possible to obtain clearer band profiles by applying a Gaussian deconvolution, resulting in a more precise identification of the molecular species present in the material, as shown in Figure 2d. The red line corresponds to the sum of the Gaussian fit and its correspondence with the experimental spectrum (white dots). Through this, a new and strong band can be detected between 800 and 900 cm^–1^ when comparing PNCB and Nb-PNCB:1. This band (in dark blue in Figure 2d) is centered at 828 cm^–1^ and shifts to 842, 851, and 859 cm^–1^ in Nb-PNCB:2, Nb-PNCB:3 and Nb-PNCB:4, respectively. According to Cardinal et al.,^52^ the broad vibrational band between 799 and 853 cm^–1^ is ascribed to the Nb–O–Nb vibration in the chains from NbO_6_ octahedra interconnected by their corners, characteristic of Nb acting as a network former.^45,52^

In contrast, the band around 900 cm^–1^ (in purple, visible in PNCB, Nb-PNCB:1 and Nb-PNCB:2) can be attributed to the stretching vibration of the Nb–O short bond in the isolated or distorted NbO_6_ octahedra.^52,53^ Therefore, the presence of NbO_6_ units is clearly confirmed by Raman spectroscopy, and the progressive shift of the band toward higher wavenumbers with increasing Nb content may reflect a structural evolution. This includes the increased formation of distorted or isolated NbO_6_ units, which may generate NBOs, suggesting a more depolymerized glass network, as indicated by ATR-FTIR, thus confirming the dual role of Nb. A more detailed description of the Raman spectra is provided in Supporting Information (Figure S4).

### 3.4 Mechanical Properties

Hardness (*H*) and elastic modulus (*E*) values of Nb glasses obtained through depth-sensing nanoindentation are displayed in Figure S5 (Supporting Information) and summarized in Table 3. The heterogeneity observed in radiopacity did not influence the mechanical properties, as the entire specimen surface was evaluated in two separate test batches. The variation in H and E as a function of the indentation load exhibits an almost linear increase of up to 100 mN for a given Nb content. However, a sharp decrease was observed at 200 mN for all samples, except for Nb-PNCB:4. Both the linear trend and the drop can be attributed to the indentation size effects. This phenomenon is associated with crack propagation underneath the indentation and the evolution of free volume during the indentation process.^54^

**Table 3:** Hardness (*H*) and Young’s Modulus (*E*) Values Measured at Different Indentation Loads in Glass Compositions of Different Nb Contents

	Load (mN)	PNCB	Nb-PNCB:1	Nb-PNCB:2	Nb-PNCB:3	Nb-PNCB:4
*H* (GPa)	10	7.6 ± 0.4	6.5 ± 0.9	7.7 ± 0.9	6 ± 1	5 ± 1
	50	12.4 ± 0.9	10 ± 1	10 ± 1	9.2 ± 0.8	9 ± 1
	100	14.6 ± 0.4	13 ± 2	13 ± 1	11 ± 2	10 ± 1
	200	10 ± 1	10 ± 1	10.6 ± 0.7	8.8 ± 0.9	11 ± 2
*E* (GPa)	10	99 ± 9	99 ± 8	104 ± 8	100 ± 8	86 ± 2
	50	126 ± 4	123 ± 10	123 ± 11	117 ± 6	106 ± 5
	100	140 ± 5	135 ± 10	131 ± 12	122 ± 9	113 ± 9
	200	117 ± 8	118 ± 8	114 ± 7	103 ± 5	117 ± 15

It is observed that the behavior of H and E exhibits a clear dependence on Nb content, which can be grouped into two regions based on their average: 1) PNCB, Nb-PNCB:1, and Nb-PNCB:2; 2) Nb-PNCB:3 and Nb-PNCB:4. In the first group, *i.e.*, glasses up to Nb-PNCB:2, demonstrated the highest H and E values, reaching an average of ∼11 and ∼119 GPa for all loads, respectively. Specifically, Nb-PNCB:1 demonstrates lower H and E compared to PNCB, while Nb-PNCB:2 maintains mechanical performance comparable to the undoped glass. This suggests that within this compositional window Nb^5+^ ions primarily function as network formers, likely entering the glass network as NbO_6_ octahedra. These units can enhance the structural integrity of the borate matrix by increasing the cross-link density, thereby preserving or even improving mechanical properties.

Beyond Nb-PNCB:2, there was a noticeable degradation in mechanical performance, where the second group (Nb-PNCB:3 and Nb-PNCB:4) indicated approximately 18 and 20% drops in H and E compared to the first group. This decline is attributed to a structural shift wherein Nb begins to act as a network modifier rather than a former. Higher Nb_2_O_5_ contents appear to facilitate the conversion of BO_4_ to BO_3_ units and promote the formation of NBOs, leading to a depolymerized network, increasing ionic mobility, reducing the glass network rigidity and mechanical properties. A similar effect has been reported by Smedskjaer et al.^40^ due to the borate unit conversion in glasses containing more than 25 mol % Na_2_O.

### 3.5 Aqueous Interactions and Ion Release

DVS was used to analyze the initial interactions of the glasses with humidity as an indicator of reactivity and potential solubility. For this purpose, gravimetric measurements of sorption were performed under direct exposure to 90% RH, as illustrated in Figure 3a.

**Figure 3:**
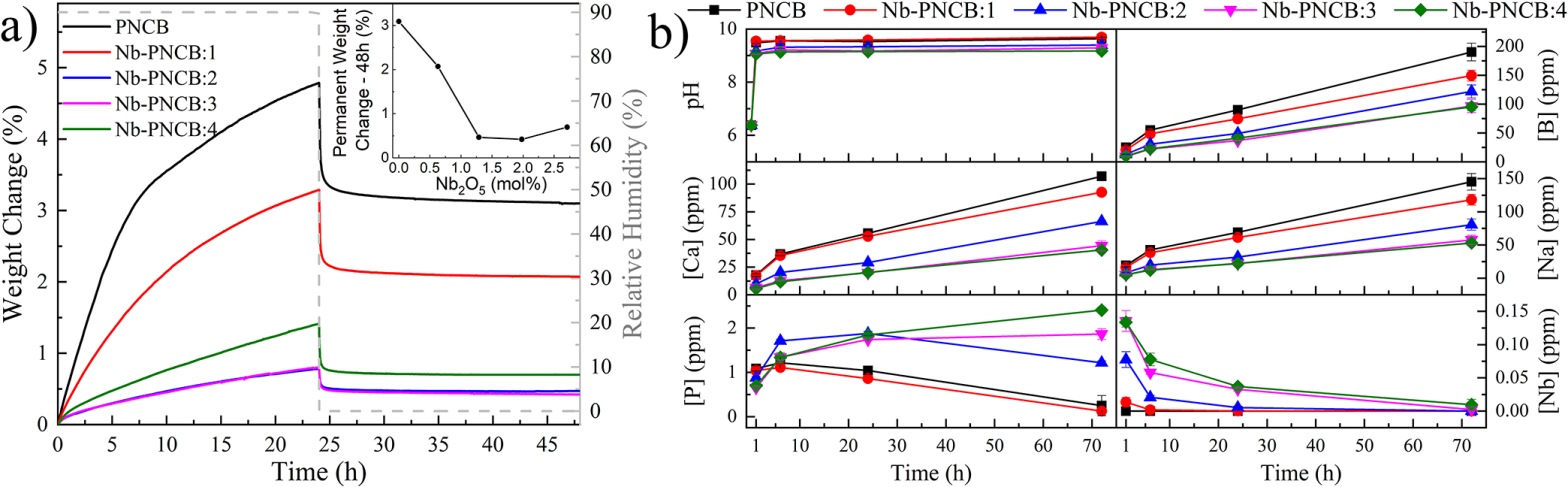
Glass aqueous interactions. a) Reactivity through vapor sorption: exposure to 90% RH followed by 0% RH. The inset shows the permanent weight change (%), related to reactivity, as a function of Nb_2_O_5_ content. b) Immersion in DIW (*n* = 3): pH measurements, and release of boron, calcium, sodium, phosphorus, and niobium ions as measured through ICP-OES.

There was an immediate and pronounced increase in the mass of PNCB during the first 6 h, attributable to vapor sorption, which was followed by a slower rate of increase until 24 h, reaching approximately 5% in total. This behavior indicates a relatively higher hydrophilicity and reactivity. As the Nb content increased, vapor sorption of the glasses decreased, demonstrating that both the rate and mass change depended on glass composition and suggesting lower extents of reactivity.

Nevertheless, Nb-PNCB:4 exhibited slightly higher reactivity (1.4%) compared to those of Nb-PNCB:2 and Nb-PNCB:3 (both at approximately 0.8%). When the RH was immediately reduced to 0%, a rapid decrease in mass was observed in all samples. After 48 h, the final mass change was higher in glasses with no or low Nb content. On the other hand, Nb-PNCB:4 exhibited a higher final mass increase than Nb-PNCB:2 and Nb-PNCB:3, as observed in the Figure 3a inset.

It is well-known that network connectivity, as well as molecular and atomic structures play critical roles in reactivity and chemical durability.^5^ In this regard, PNCB presented a higher reactivity. When Nb_2_O_5_ is incorporated in the glass, Nb^5+^ ions primarily form NbO_6_ octahedra that incorporate into the borate network and act as network formers, thereby increasing the overall cross-link density. As a result, the glass becomes more chemically durable and less susceptible to moisture-induced degradation, as evidenced by the reduced water sorption observed in Nb-PNCB:2 and Nb-PNCB:3.

However, for Nb-PNCB:4, the glass network exhibits signs of depolymerization. ATR-FTIR spectra indicated an increase in BO_3_ units and a corresponding decrease in BO_4_ units, suggesting a shift toward a less connected structure. Raman spectroscopy further revealed changes in vibrational modes associated with Nb–O bonds, indicating alterations in the NbO_6_ environment. These structural modifications likely introduce more NBOs, increasing the free volume and facilitating a higher water interaction, as evidenced by the higher residual mass gain in Nb-PNCB:4 after RH reduction.

To better understand the effect of Nb content on reactivity and to explore ion release kinetics, the release of boron, calcium, sodium, phosphorus, and niobium during glass dissolution was measured for up to 72 h in DIW using ICP-OES, along with changes in its pH (Figure 3b).

Initially, it was observed that the pH of the aqueous solution increased significantly within the first hour of immersion, which was followed by a slight increase up to 6 h and remaining stable until 72 h. It is known that the hydration reaction may result in the exchange of cations (such as Na^+^, Ca^2+^, or others) with the solution and the consumption of H^+^, while the hydrolysis reaction increases the pH of the solution due to the release of OH^–^.^6,9^ This suggests that, before 6 h, hydrolysis was more dominant, beyond which both hydration and hydrolysis reactions occurred at a similar rate.^6^

PNCB and Nb-PNCB:1 showed slightly higher pH values compared to Nb-PNCB:2, Nb-PNCB:3 and Nb-PNCB:4. From a chemical perspective, the modifier role of Nb at higher concentrations enhances ionic mobility within the glass, thus facilitating hydrolysis and ion exchange reactions at the glass-solution interface. This is supported by DVS data, showing increased water uptake for Nb-PNCB:4. The reactivity is also reflected in the pH trends during immersion tests, where these compositions show a less pronounced increase in pH compared to low Nb-content glasses and is consistent with altered hydration and hydrolysis dynamics. Similar observations have been made on pH values for Nb-doped silicate glasses.^15^

The release of boron, calcium, and sodium was gradual, with glasses of higher Nb content resulting in lower extents of release. On the other hand, phosphorus and niobium indicated distinct release profiles, with phosphorus demonstrating a decreasing trend after 6 h for PNCB and Nb-PNCB:1, and after 24 h for Nb-PNCB:2. For Nb-PNCB:3 and Nb-PNCB:4, there was an increasing trend over time up to 72 h. For Nb, the highest release was detected during the first hour, followed by its reduction, until it was no longer detectable by 72 h.

Although the concentration of boron increases in glasses with a higher Nb content, the effect of this increase was not detectable in its ionic release measurements. It is reported that glasses rich in BO_4_ typically exhibit lower dissolution rates, especially in those produced via melt-quenching, as BO_4_ increases the network connectivity.^6,55^ However, in binary CaO-B_2_O_3_ glasses, it has been reported that higher N_4_ contents, release ions more quickly,^5^ which could be due to the presence of network modifiers and interactions with other components, such as niobium.^15^ Recently, Yin et al.^6^ studied the reactivity mechanism in the dissolution and reactions of various calcium-borate glasses and concluded that the initial release rates of calcium and boron are mainly influenced by their respective concentrations in the material, rather than the N_4_ ratio.^6^

The release of calcium and sodium was consistent with expected trends as niobium is added at the expense of these ions, whereas the release of phosphorus decreased over time, likely due to its consumption, possibly as a result of interaction with calcium. This interaction is commonly observed in bioactive glasses, and when in physiological medium, such as SBF, forms a surface HCA (hydroxycarbonate apatite) layer.^5,15^ However, for Nb-PNCB:3 and Nb-PNCB:4, this reduction in phosphorus was not observed, suggesting a possible interference of niobium in the kinetics of HCA layer formation. Niobium may influence the surface reactivity of the glasses, modifying the dissolution rate of phosphate ions or inhibiting the nucleation of the apatite phase.^15^

Nb release was found to increase, in accordance with its content in the glasses. In the first hour, this is detected due to the leaching of niobium species near the surface and the possible release of isolated NbO_6_ units.^16^ However, niobium can interact with phosphate and hydroxyl groups in solution, forming Nb–O–P or Nb–OH.^15,16^ There are also reports of the formation of calcium niobate and/or sodium niobate species.^17^ Consequently, the concentration of Nb-free glass decreases in the solution and may precipitate on the glass surface as a niobium gel, limiting the reactivity. Thus, the rate at which niobium species are removed from solution is gradual and can be mainly attributed to their polymerization.^15^ This is in agreement with the pH values and mass change, as observed by DVS.

### 3.6 Acelullar Bioactivity in SBF

The *in vitro* bioactivity of the Nb-incorporated glasses was evaluated in SBF for up to 14 days. Figure 4a shows the XRD patterns obtained for each immersion time. For the PNCB glass, the formation of HCA was detected as early as day 3, as evidenced by the appearance of sharp diffraction peaks at 2θ = 26° and 32°, which correspond to the (002) and (211) planes of hydroxyapatite, respectively (JCPDS 9-0432).^56^ The intensity of these peaks increased with immersion time, indicating progressive crystallization.

**Figure 4:**
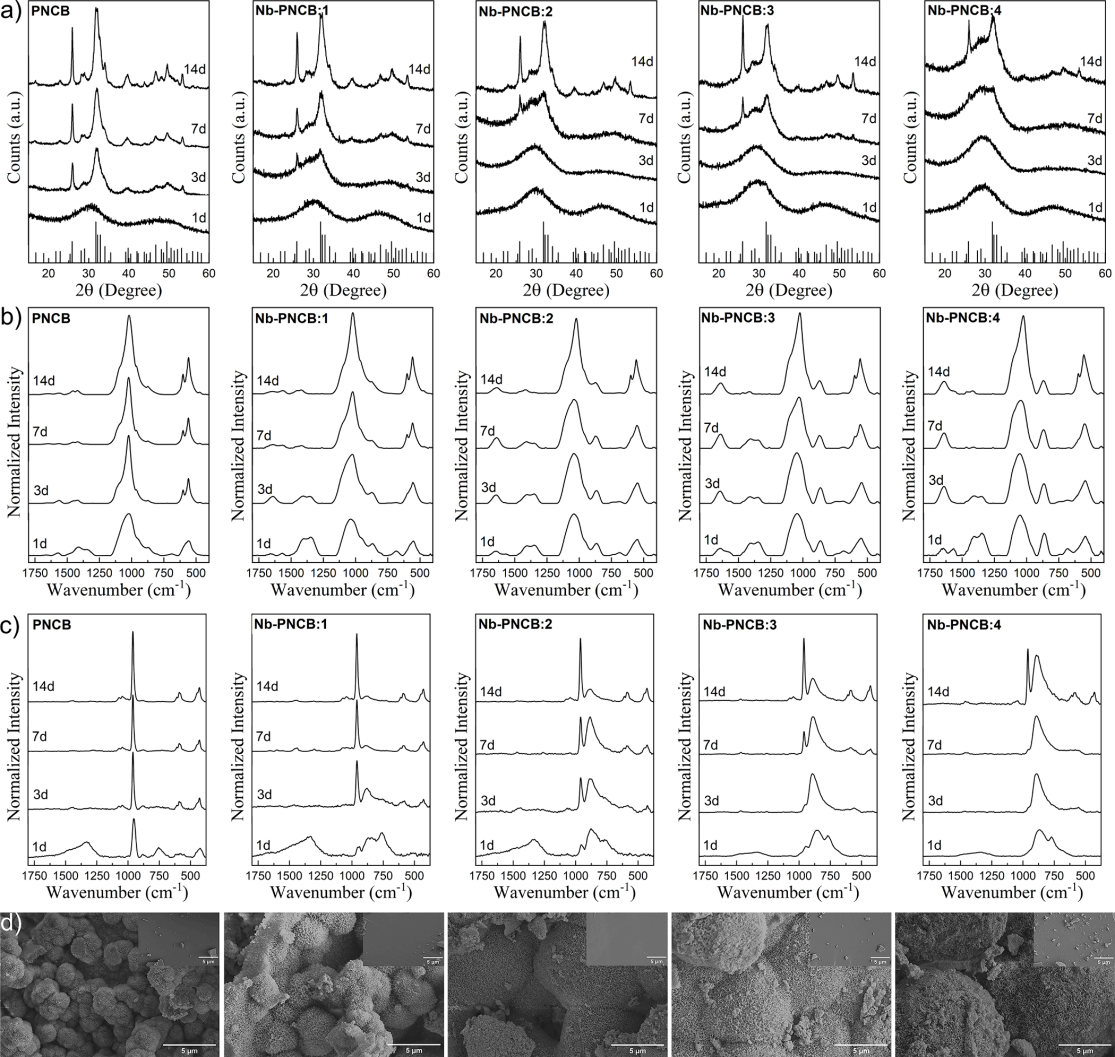
Acellular bioactivity of PNCB and Nb-PNCB immersed in SBF. a) XRD diffractograms after 1, 3, 7, and 14 days. The indexed phase corresponds to hydroxyapatite JCPDS 9-0432. b) ATR-FTIR spectra after 1, 3, 7, and 14 days. c) Raman spectra after 1, 3, 7, and 14 days. d) SEM images of the glasses after 14 days, and the inset are images before immersion.

On the other hand, as the Nb content increased, HCA conversion occurred at progressively slower rates. For Nb-PNCB:1, the first signal of well-defined apatite peaks appeared after 3 days, while for Nb-PNCB:2 and Nb-PNCB:3, they became noticeable at day 7. In the case of Nb-PNCB:4, no sharp peaks were detected until between days 7 and 14, and even then, the bands remained relatively broad, suggesting partial crystallinity and the persistence of an amorphous phase.^56^

ATR-FTIR spectra of the glasses as a function of time in SBF confirmed this conversion through the appearance of characteristic bands in the HCA layer (Figure 4b). These bands included the ν_3_ (asymmetric stretching) and ν_1_ (symmetric stretching) peaks of phosphate (PO_4_^3–^) at 1020 cm^–1^ and the shoulder at 961 cm^–1^. Additionally, the ν_4_ of phosphate was also observed between 550 and 600 cm^–1^, while the band at 1640 cm^–1^ can be attributed to OH group vibrations. Furthermore, the substitution of OH^–^ sites (Type A substitution) or PO_4_^3–^ sites (Type B substitution) by carbonate (CO_3_^2–^) can occur in the hydroxyapatite structure, leading to the formation of HCA, characterized by the ν_3_ values of CO_3_^2–^ at 1420 and 1457 cm^–1^. It is worth noting that the splitting in the ν_4_ phosphate bands indicates layer crystallization.^5,56–58^ Thus, all glasses formed an amorphous layer on the first day of immersion, and this layer crystallized more rapidly in PNCB (within 3 days), followed by Nb-PNCB:1 (within 7 days), and finally Nb-PNCB:2, Nb-PNCB:3, and Nb-PNCB:4 (detectable at 14 days).

Raman spectroscopy (Figure 4c) confirmed the progressive formation of a HCA layer on the glass surfaces, as evidenced by the emergence of characteristic phosphate bands. The most prominent peak corresponds to the symmetric stretching mode (ν_1_) of PO_4_^3–^ at approximately 961 cm^–1^, while additional phosphate-related bands were observed between 430 and 450 cm^–1^ (ν_2_), 570 and 610 cm^–1^ (ν_4_), and 1020–1045 cm^–1^ (ν_3_). Furthermore, carbonate incorporation into the apatite lattice was indicated by the presence of the ν_1_ band of CO_3_^2–^ between 1070 and 1090 cm^–1^ and the ν_3_ mode near 1450 cm^–1^.^59,60^ These spectra corroborated the XRD findings, indicating that a HCA layer initially formed on the first day of immersion for PNCB, followed by progressive crystallization, as detected by narrowing of the peaks. In contrast, the initial spectra of Nb-incorporated glasses resembled those of the as-prepared samples, indicating no significant structural conversion. However, with longer immersion times, the spectra gradually evolved, evidencing the formation of a HCA layer.

Interestingly, beyond the phosphate and carbonate signals, an additional Raman feature appears in the range of 865–885 cm^–1^ after immersion in SBF, becoming more evident at glasses with higher Nb contest. This band can be attributed to Nb–O vibrational modes,^52^ suggesting an enrichment of Nb at the glass surface during the dissolution–precipitation process. These observations are consistent with the EDS (Figure S6) results, which indicate an increase in the Nb content on the glass surface after SBF immersion. Furthermore, the Ca/P ratio changed between the samples, possibly related to the influence of niobium on the nucleation and growth kinetics of the apatitic layer.

SEM images were obtained to examine the morphological changes on the surface of the glasses after 14 days of immersion in SBF (Figure 4d). For the PNCB, spherical aggregates characteristic of calcium phosphate (CaP) coatings are observed, typical of the precipitates formed during the reaction with SBF.^61^ With the incorporation of Nb, a slight change in the surface morphology is noted, with the diameters of the formed spheres being slightly larger and regions with more irregular textures, such as crystals, exhibiting rough and heterogeneous areas, behavior similar to that described by Ferreira et al. for bioactive calcium niobate particles obtained by the sol–gel method.^62^

Overall, these results show that glasses with a higher Nb_2_O_5_ content exhibited lower HCA conversion rates. This is consistent with previous studies reporting that increasing Nb_2_O_5_ content in silicate glasses demonstrated decreasing *in vitro* mineralization rates.^15^ The reduction in the dissolution rate may have led to a lower conversion rate, possibly due to the formation of an Nb–OH layer, which slows down the precipitation of calcium and phosphorus on the glass surface.^15,16^ This phenomenon is also indicated by the lack of phosphorus consumption during the first 7 days in DIW for Nb-PNCB:3 and Nb-PNCB:4.

Additionally, in some silicate glass compositions, ion release is delayed due to the formation of a SiO_2_-rich layer.^9,44,63^ However, borate glasses do not exhibit this capability, suggesting that HCA initially forms on the outer surface of the glass and continuously reacts toward the center, causing a decrease in volume until complete conversion. This process is associated with the relatively rapid release of BO_3_^3–^ and Na^+^ ions, while Ca^2+^ and PO_4_^3–^ ions migrate to the surface,^44,63^ leading to the formation of an amorphous calcium phosphate layer that ultimately crystallizes into HCA.

### 3.7 Cellular Assays

Figure 5a shows a matrix of fluorescence microscopy images obtained over 10 days of huAD-MSC culturing in the presence of medium only (control group) and those treated with ionic release products from PNCB, Nb-PNCB:1 and Nb-PNCB:4. Cells were stained with fluorescent markers, where the cytoskeleton (actin) appears green and the nuclei blue (DAPI), allowing observation of viable cell morphology and density over time, while dead cells were stained red (DNA). All cultures exhibited high viability and cellular density through time, as indicated by green cells, with minimum presence of dead cells. There was a progressive increase in cell density from days 1 to 10 under all conditions, indicating cell proliferation. Based on morphological analysis, cells showed their characteristic spindle-like shape, indicating no differences in cell morphology when compared with control samples. This suggests that the presence of Nb did not affect the expansion, viability and morphology of the MSCs.

**Figure 5:**
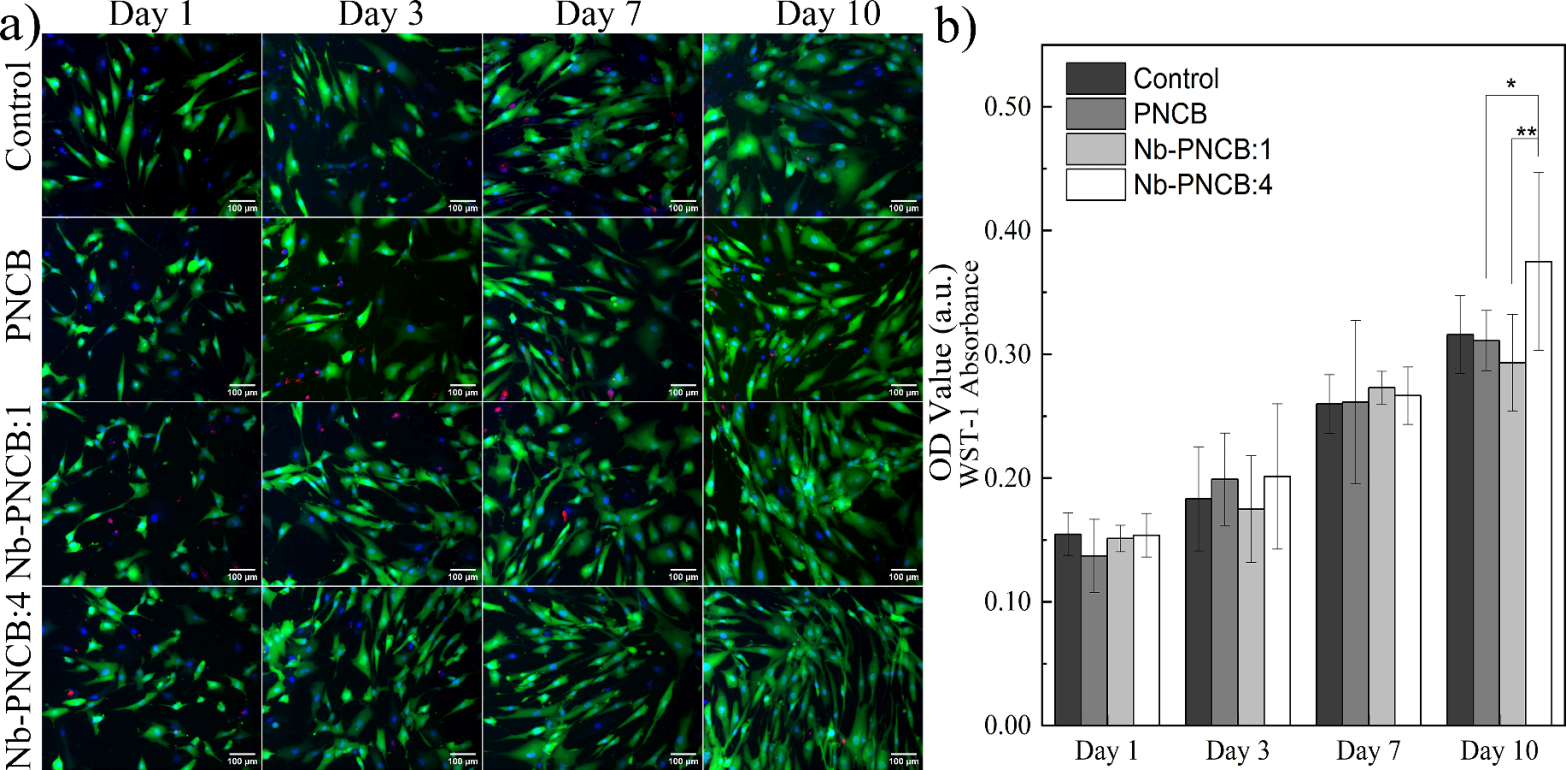
Cellular assays. a) Calcein-AM labeled live huAD-MSC (green), Hoechst 33342 (blue) stained dsDNA, and Ethidium Homodimer-1 binding dead nuclei (red) at days 1, 3, 7, and 10. Scale bar = 100 μm. b) Cell metabolic activity assessed by absorbance. All experiments were treated with culture supplemented with ionic dissolution products of PNCB, Nb-PNCB:1, and Nb-PNCB:4 at a concentration of 1.5 mg/mL. Statistical analysis was performed using two-way ANOVA followed by Tukey’s posthoc test. Significant differences are indicated as follows: *p < 0.05, **p < 0.01.

The samples were also subjected to the WST-1 metabolic assay (Figure 5b), which evaluated cell viability based on mitochondrial activity, indicating the proliferative potential of the cells. The results indicated a progressive increase in optical density over time, suggesting increased metabolic activity. No significant differences were found at earlier stages of culture, but on day 10, Nb-PNCB:4 exhibited relatively higher metabolic activity (*p* < 0.05 for PNCB and *p* < 0.01 for Nb-PNCB:1).

It is well established that the pH of the culture medium directly affects cell viability and proliferation.^64–66^ The DVS analysis indicated that PNCB exhibited higher reactivity compared to Nb-PNCB:1 and Nb-PNCB:4 (Figure 3a), which directly impacts their ionic release (Figure 3b). Lower ion release rates are associated with reduced pH values, bringing them closer to physiological pH. Nevertheless, these results suggest that the addition of niobium to borate glass did not negatively alter the viability and proliferation of MSCs. On the contrary, Nb incorporation promoted cell proliferation when the ionic dissolution products of the glasses were added to the culture medium. A similar outcome has been observed for silicate glasses containing up to 2.7 mol % Nb_2_O_5,_ which showed no cytotoxicity but also did not significantly stimulate cell proliferation, as assessed by metabolic assays using human embryonic stem cells (hESCs).^17^ These findings are also in agreement with a study by Miguez-Pacheco et al.,^1^ who reported enhanced proliferation of bone marrow stromal cells in response to dissolution products of silicate glasses containing up to 1 mol % Nb_2_O_5_, with the most pronounced stimulatory effect observed at intermediate dilution concentrations (0.1 mg/mL).^1^

The addition of niobium to phosphate glasses has also led to a reduction in ion release rates and, consequently, decreased the acidification of the medium (a typical effect on solution pH by phosphate-based glasses) thereby promoting greater cell proliferation even in glasses with up to 30 mol % Nb_2_O_5_.^67^ Calcium phosphate invert glasses containing up to 10 mol % Nb_2_O_5_ also demonstrated cytocompatibility, upregulation of alkaline phosphatase activity and enhanced calcium deposition in 3 and 5 mol % Nb_2_O_5_, even in the absence of osteogenic supplements.^4^

Additionally, Lopes et al.^16^ evaluated the responses of bone marrow-derived MSCs to silicate glasses exposed to conditioned media containing ionic dissolution products of 45S5-based glasses doped with up to 5 mol % Nb_2_O_5_. Cell viability assays showed no toxicity, with the 1 mol % Nb_2_O_5_ group promoting the highest cell proliferation. Compositions containing 1 and 2.5 mol % induced osteogenic differentiation, as evidenced by the formation of mineralized nodules and osteocalcin expression. These observations suggest that the dissolution products of these compositions support osteoblastic maturation, even without specific chemical stimuli. Moreover, *in vivo* tests demonstrated that the 1 mol % glass was osteoconductive, promoting bone formation around the implant in rat tibiae.^16^

Therefore, in Nb-incorporated borate glasses, the incorporated amount and the concentration selected for the dissolution products are consistent with previous reports and align well with the latest findings in the literature. The observed enhancement in cell proliferation and cytocompatibility corroborates studies on different glass systems containing Nb_2_O_5_, where similar trends have been reported and support cell development and cytocompatibility.^1,4,16,17,68^ These results collectively support the potential of Nb-incorporated borate glasses to promote a favorable cellular environment.

### 3.8 Blood Coagulation

Figure 6a shows the shear storage modulus (*G*′) curves related to clot formation in citrated blood exposed to different concentrations of ionic dissolution products and particles from PNCB and Nb-PNCB:4.

**Figure 6:**
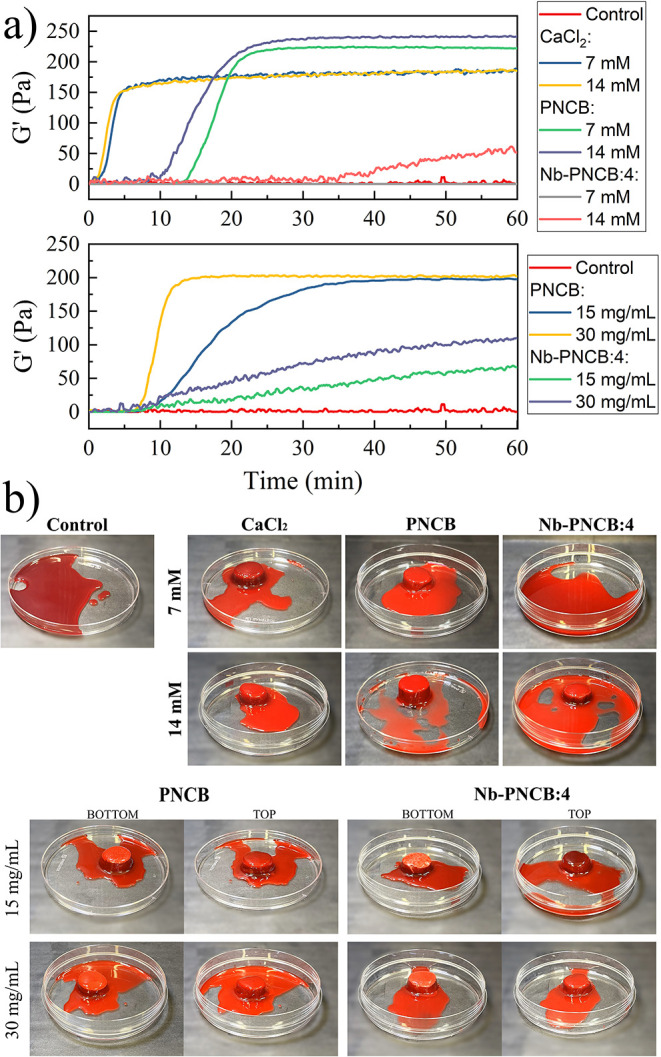
Clotting profiles. a) *G*′ versus time curves of citrated blood mixed with ionic dissolution products of PNCB and Nb-PNCB:4 (top) and their particles (bottom). b) Photographic images of citrated blood at the end of tests mixed with 7 and 14 mM ionic dissolution products of PNCB and Nb-PNCB:4 and glass particles at 15 and 30 mg/mL.

It was observed that the negative control showed no tendency toward coagulation with no change in *G*′ demonstrated throughout the entire analysis period. In contrast, the positive controls with 7 and 14 mM CaCl_2_ exhibited similar behaviors, with initiation times of 1.2 ± 0.1 and 1.1 ± 0.2 min, and stabilization times of 4.4 ± 0.1 and 3.8 ± 0.1 min, respectively. After 60 min, the *G*′ values reached 178 ± 2 and 179 ± 2 Pa, respectively.

For PNCB, longer times were observed for the onset of clot formation compared to the controls, *i.e.*, at 12 ± 1 and 9 ± 1 min, with stabilization times of 22 ± 2 and 20.3 ± 0.4 min for 7 and 14 mM, respectively. However, these groups exhibited higher *G*′ values, reaching 223 ± 2 and 235 ± 5 Pa, respectively. In the case of Nb-PNCB:4, the 7 mM concentration was not sufficient to induce clot formation, while at 14 mM, only a small clot was formed. Under this condition, coagulation began at 35 ± 2 min.

This behavior was also reproduced when the blood was directly exposed to glass particles, where the 30 mg/mL concentration led to better clot formation compared to 15 mg/mL. However, PNCB promoted better clot formation, with *G*′ increasing significantly compared to Nb-PNCB:4. For PNCB at 15 mg/mL, clot initiation was detected at 7 ± 3 min, with stabilization at 28 ± 3 min and a final *G*′ of 199 ± 8 Pa. At 30 mg/mL, clotting began at 5.8 ± 0.7 min and stabilized at 11 ± 1 min, reaching a final *G*′ value of 200 ± 2 Pa. For Nb-PNCB:4, the initiation times were 6 ± 1 min for 15 mg/mL and 4.4 ± 0.8 min for 30 mg/mL. However, no curve stabilization was observed within 60 min, suggesting incomplete clot formation. All values are summarized in Table 4.

**Table 4:** Clot Initiation Time, Clot Stabilization Time, and Final Storage Modulus (*G*′) Values Were Obtained When Citrated Blood Was Exposed to Ionic Release (CaCl_2_, PNCB, Nb-PNCB:4) or Particles (PNCB, Nb-PNCB:4)

	Ion release (mM)/Particles (mg/mL)	Clot initiation time (min)	Clot stabilization time (min)	*G*′ stabilized (Pa)
CaCl_2_	7 mM	1.2 ± 0.1	4.4 ± 0.1	178 ± 2
	14 mM	1.1 ± 0.2	3.8 ± 0.1	179 ± 2
PNCB	7 mM	12 ± 1	22 ± 2	223 ± 2
	14 mM	9 ± 1	20.3 ± 0.4	235 ± 5
Nb-PNCB:4	7 mM	-	-	-
	14 mM	35 ± 2	-	-
PNCB	15 mg/mL	7 ± 3	28 ± 3	199 ± 8
	30 mg/mL	5.8 ± 0.7	11 ± 1	200 ± 2
Nb-PNCB:4	15 mg/mL	6 ± 1	-	-
	30 mg/mL	4.4 ± 0.8	-	-

Photographic images (Figure 6b) complement the data, with the negative control appearing as a homogeneous fluid and no sign of coagulation, whereas the positive CaCl_2_ control formed a well-defined clot. In PNCB, clot formation was dense, with few areas showing fluid blood and 14 mM displaying lower flow. In contrast, Nb-PNCB:4 presented limited clot formation, with no clot observed at 7 mM and a high fluidity at 14 mM, indicating reduced conversion of fibrinogen into fibrin.

This pattern was also evident in the suspensions containing glass particles. Clots formed in the presence of PNCB produced more compact and cohesive structures compared to Nb-PNCB:4, visible from both the top and bottom views of the clots, where the particles settled.

Although CaCl_2_ triggered a faster response, PNCB achieved higher *G*′ values, suggesting a more elastic response, potentially influenced not only by the released calcium but also by the contribution of other ions present in the composition. There was a dose-dependent effect: the higher the ion or particle concentration, the higher the *G*′ value. However, the presence of niobium impaired clot formation, which may be explained by the lower calcium content and release (Table 1 and Figure 3b) or by the presence of niobate complexes, as discussed above. Thus, both the released ions and the physicochemical interaction of the particle surfaces appear to play direct roles in the response with blood components.

Blood coagulation or hemostasis occurs in three main stages: vasoconstriction, formation of the platelet plug, and the actual coagulation process. In the final phase, known as the coagulation cascade, a series of plasma proteins called clotting factors is activated in sequence, leading to the conversion of fibrinogen into fibrin. In this stage, Ca^2+^ ions play a crucial role, as they are required to activate factors such as II (prothrombin), VII, IX, and X. Calcium is also essential for the formation of the tenase and prothrombinase complexes.^33,69^ Although Na^+^ ions are not directly involved in the coagulation cascade, they are important for maintaining electrolyte balance and proper cellular function, including that of platelets.^69^ More widely, other ions such as potassium and zinc have attracted research interest for their potential roles in process.^70^ Thus, blood coagulation is a highly coordinated mechanism that depends not only on the interaction between proteins and cells but also on a balanced presence of ions for it to occur efficiently.^8,34,69^ In this regard, high *G*′ values and rapid increases are associated with fibrin network formation and stabilization, reflecting the transition of blood from the liquid state to a gel.

These findings are consistent with the results reported by Naseri et al.,^34^ who observed a concentration-dependent behavior for the CaCl_2_. Similarly, Rezabeigi et al.^33^ not only demonstrated the efficiency of the technique but also characterized different hemostatic agents and their potential to affect hemostasis, depending on the specific mechanisms of each material.^33^ Therefore, the response observed in this study also suggests that it is possible to modulate the hemostatic response by tuning the glass composition, ionic release, and particle concentration, depending on the intended use. This versatility makes bioactive borate glasses promising candidates for use as local hemostatic agents, especially in clinical scenarios requiring rapid bleeding control, such as in surgeries or traumatic wounds, as discussed by Pourshahrestani et al.^8^

Therefore, while the incorporation of Nb into borate glass networks has been previously reported, this study provides novel insights into how the resulting structural modifications influence a cascade of material properties, mainly bioactivity and cellular responses. The dual role of Nb demonstrates that even subtle changes in network connectivity can have pronounced multifunctional effects. Collectively, these findings emphasize that understanding the structure–property relationships of Nb-incorporated borate glasses enables rational strategies to design bioactive glasses with tailored, multifunctional properties for biomedical applications.

## 4 Conclusion

The dual role of Nb as both a network former and a modifier in bioactive borate glasses significantly influences their structural, thermal, mechanical, and chemical properties. At low concentrations, Nb^5+^ ions contribute to increased glass transition temperatures and enhanced network connectivity by forming NbO_6_ octahedra that bridge the glass network. However, higher Nb_2_O_5_ contents lead to increased NBOs, decreased network connectivity, and reduced packing density, resulting in lower *T*_g_ and diminished mechanical properties such as hardness and elastic modulus.

Although the structural role of Nb in the vitreous network has already been studied, even at higher concentrations, its relationship with bioactivity and biocompatibility assays has not yet been considered.

In terms of ionic release and HCA formation, the increase in Nb content led to a reduction in reactivity and ionic release, which, in turn, slowed down the formation of the apatite layer. On the other hand, Nb enhanced cellular viability, although the underlying mechanisms are yet to be fully understood. Interestingly, Nb interfered with blood coagulation, possibly due to the formation of Nb-complexes. Nevertheless, dose-dependent effects were observed, and Nb-free bioactive borate glasses may be promising hemostatic agents.

In summary, the results demonstrate that Nb exhibits a dual structural role in bioactive borate glasses, contributing to both network formation and modification. This duality imparts tunable properties that are particularly advantageous for biomedical applications.

## Supplementary Material



## Notes

X-ray fluorescence (Table S1); photographic images of glasses (Figure S1); radiographic image (Figure S2); thermograms and diffractograms (Figure S3a and S3b); Raman discussion and spectra (Figure S4a and S4b); hardness and elastic modulus graphs (Figure S5); energy-dispersive X-ray spectroscopy (EDS) spectra (Figure S6) (PDF)
