# Supplementary material for: Dual Structural Role of Niobium in Bioactive Borate Glasses Modulates Bioactivity, Cytocompatibility, and Hemostatic Potential
Source: ACS Appl Mater Interfaces. 2025 Oct 24;17(44):60213–26. doi: 10.1021/acsami.5c13713 (PMC12598716; doi:10.1021/acsami.5c13713)
Supplement: Supplementary file 1 [file am5c13713_si_001.pdf]

Supporting Information

Dual structural role of niobium in bioactive borate glasses modulates bioactivity, cytocompatibility, and hemostatic potential

Mariana Sversut Gibbin[ab*], Vitor Santaella Zanuto[a], Jose G. Munguia-Lopez[bc], Robson Ferrari Muniz[a], Pierre Hudon[b], Alejandra Islas Encalada[b], Richard R. Chromik[b], Francielle Sato[a], Showan N. Nazhat[b*].

[a] Departamento de Física, Universidade Estadual de Maringá, Maringá, PR, 87020-900, Brazil

[b] Department of Mining and Materials Engineering, McGill University, Montreal, QC, H3A 2A7, Canada

[c] Department of Bioengineering, McGill University, Montreal, QC, H3A 2A7, Canada

* Corresponding Authors: Mariana Sversut Gibbin (marigibin32@gmail.com); Showan N. Nazhat (showan.nazhat@mcgill.ca).

Table S1: Chemical composition (in wt%) of the glasses estimated by X-ray fluorescence spectroscopy (XRF).

Sample	B_2O_3	CaO	Na_2O	P_2O_5	Nb_2O_5
**PNCB**	60.00	17.93	20.46	1.62	0.00
**Nb-PNCB:1**	60.00	16.27	20.37	1.56	1.80
**Nb-PNCB:2**	60.00	14.30	19.74	1.60	4.37
**Nb-PNCB:3**	60.00	12.89	18.97	1.63	6.50
**Nb-PNCB:4**	60.00	14.43	14.43	2.05	8.52

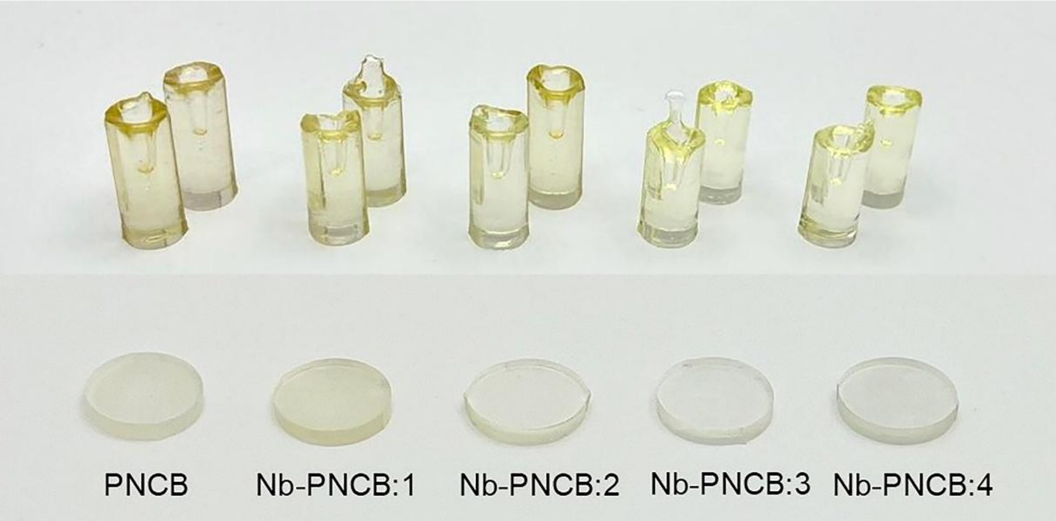

Figure S1: Photographic images of as-made Nb-incorporated borate glasses.

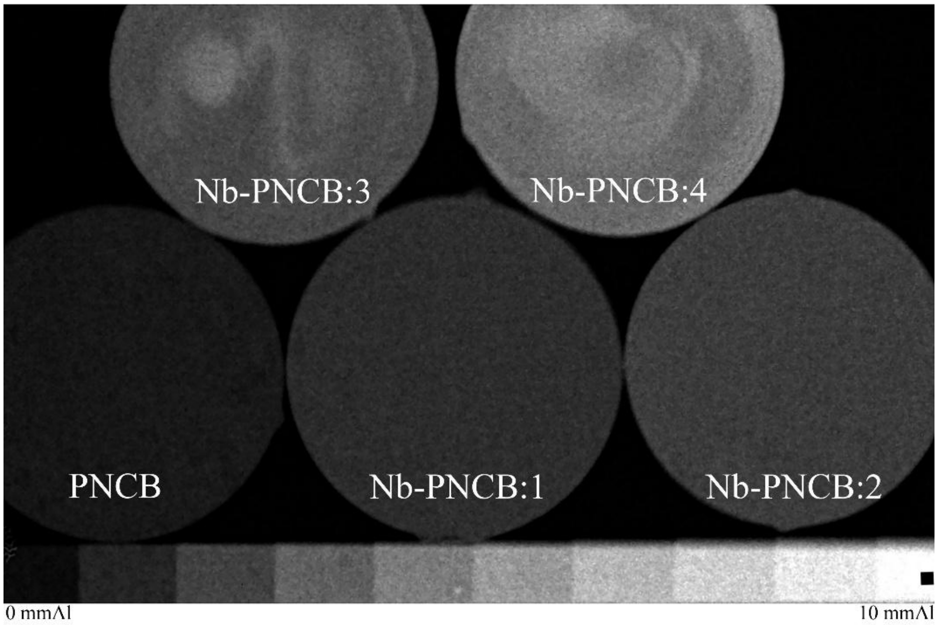

Figure S2: Radiographic images of as-made Nb-incorporated borate glasses.

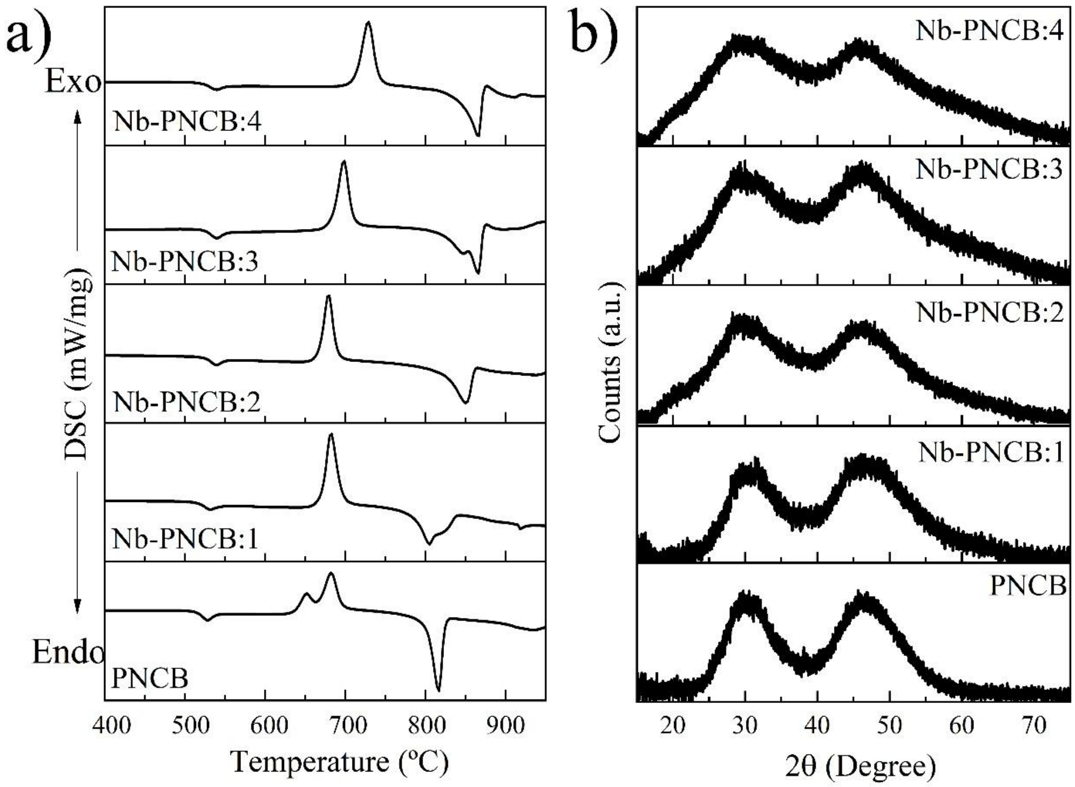

Figure S3: a) DSC thermograms, and b) XRD diffractograms of Nb-incorporated borate glasses.

RAMAN DISCUSSION:

Borate glasses are significantly altered through the incorporation of alkaline earth oxides, resulting in the formation of more complex structures, such as polyborate groups. Raman spectroscopy has been shown to be a successful method for discerning the characteristics of these groups [1,2]. The concentration of these borate groups in each glass composition depends on the nature and proportion of network-forming and network-modifying oxides [3]. Figures 2c and 2d shows the Raman spectra of the Nb-incorporated borate glasses developed this study. Figure S4a and S4b (which is identical to Figure 2c and 2d) was added for easy reading.

Borate arrangements containing $BØ_4$ tetrahedra may be identified due to presence of the bands around 1131 and 526 cm^{-1} [1,4–6], which are related to diborate units, whether connected or isolated, respectively [5,6] (Figure 2c/S4a). When the Nb content is increased, a reduction in this vibrational mode can be detected and consequently, it may be related to the decrease in bridging oxygens [1]. According to Yiannopoulos *et al.*, the band at 1475 cm^{-1} may be associated with the stretching of the terminal $B–O^-$ bonds in $BØ_2O^-$, mainly attached to the borate network segment [1,3–5]. Breaking these connections can cause a reduction in band intensity. Furthermore, depending on modifications caused by the concentrations of alkali and alkaline earth oxides, a slight shift of these bands may be exhibited in agreement with Konijnendijk and Stevels [2].

The region between 1050 and 600 cm^{-1} is characterized by an overlap of vibrational modes. To decompose these into individual components, Gaussian deconvolution has proven to be effective as it aims to model and isolate the specific contributions of each band, allowing a more detailed analysis, particularly in situations where there is overlapping, making it challenging to conduct a precise and specific analysis.

By applying deconvolution, it is possible to obtain clearer band profiles, resulting in a more precise identification of the molecular species present in the glasses, as shown in Figure 2d/S4b). The red line corresponds to the sum of gaussian fit and its correspondence with the experimental spectrum (white dots). The cyan band centered at 648 cm^{-1} for PNCB is attributed to metaborate groups, specifically the fraction of $BØ_2O^-$ triangles, which is arranged in metaborate rings ($B_3O_6^{3-}$) and manifested as a result of symmetric breathing vibration [1,4–6], where O is associated with NBOs (non-bridging oxygens) and Ø with BOs (bridging oxygens). In Nb-incorporated glasses, this band moves up to 703 cm^{-1} and increases the area linearly (Nb-PNCB:4). A broad band can be detected at 652–659 cm^{-1} and ascribed to the Nb–O–Nb vibrations of NbO_6 octahedra shared by vertices [7–9]. Basically, this was expected due to the linear increase of Nb_2O_5.

The green band, centered at 742 cm^{-1}, is associated with the vibrational modes of metaborate chains [1,2,6]. It tends to shift to lower wavenumbers, and its area decreases with niobium incorporation, though not extinguished. The band centered at 766 cm^{-1} in PNCB, highlighted by the pink curve, is attributed to superstructures formed by two six-membered rings interconnected by two $BØ_4^-$ tetrahedra [1,2,5,6,10,11]. According to Konijnendijk and Stevels, the detection of a peak close to 760 cm^{-1} indicates the existence of a notable quantity of $BØ_4$ units [2]. However, with niobium incorporation, this shifted to higher wavenumbers, *i,e.,* up to 770 cm^{-1} for Nb-PNCB:4, suggesting a partial conversion tendency from one $BØ_4^-$ tetrahedron to one $BØ_3$ triangle [4,11]. The opposite has been observed in $BaO–B_2O_3$ systems [4]. The band close to 774 cm^{-1} may be assigned to the symmetric breathing vibration of six membered rings with one $BØ_4^-$ tetrahedron. The gradual broadening with Ba addition and shifting to lower frequencies band suggests the formation of six membered rings with two $BØ_4^-$ tetrahedra [4].

Moreover, the olive curve at 985 cm^{-1} for PNCB is associated with the presence of diborate groups [1,12]. This band slightly shifts up to 979 cm^{-1} for Nb-PNCB:4, but the area and width tend to remain constant. The association between borate and phosphate groups can generate B–O–P bonds represented by the orange curve centered at 943 cm^{-1} for PNCB. The area associated with this band decreases until its complete disappearance in Nb-PNCB:4 and may be associated with Nb_2O_5 addition, as a new and strong band (in dark blue) appears between 800 and 900 cm^{-1}. For Nb-PNCB:1, the band is centered at 828 cm^{-1} and for Nb-PNCB:2, Nb-PNCB:3 and Nb-PNCB:4, it shifts to 842, 851 and 859 cm^{-1}, respectively. According to Cardinal *et al.*, a broad vibrational band in the range 799–853 cm^{-1} can be ascribed to the Nb–O–Nb vibration in the chains from NbO_6 octahedra interconnected by their corners [9].

The purple curve at approximately 916 cm^{-1} for PNCB, shifts to 902 cm^{-1} for Nb-PNCB:2. In glasses with higher concentrations of Nb, this disappears once it is no longer necessary to fit the experimental curve. While no clear assignment is offered for this band, it may be associated with asymmetric vibration of "loose" $BØ_4$ tetrahedra, according to Kamitsos and Chryssikos, as based on a $Cs_2O–B_2O_3$ system [10] or even pentaborate structures [13]. However, the band around 900 cm^{-1} could be also identified as the stretching vibration of the Nb–O short bond in the isolated, distorted NbO_6 octahedra [7,9] or even, may present NBOs [14]. Therefore, the reduction of the bands at 943 cm^{-1} and the shift from 842 cm^{-1} to higher wavenumbers can be linked to the possibility of substitutions such as B–O–Nb[15] and includes the increased formation of distorted or isolated NbO_6 units, which may generate NBOs, indicating a more depolymerized glass network.

The distortion of borate structures in the glasses is substantial, indicating variability in bond angles and/or lengths of the B-O bonds within each borate group. The main change associated with this is the slight shift in peak wavenumbers, as well as the variations in FMWH and area. This distortion is not uniform across all borate groups and the Raman spectra does not provide clarity on the specific number of NBOs formed in association with the borate groups[2], providing only a general overview of what may be occurring in the glass. Nevertheless, the Raman spectra have provided evidence of Nb incorporation in the glasses, which was responsible for reducing the diborate structures and connecting to metaborate rings and chains, since its vibrational modes had its intensity decreased, forming a tridimensional network, mainly based in NbO_6 octahedra. Furthermore, $BØ_4$ tetrahedra was reduced, as also indicated through FTIR (Figure 2a&b), and possible substitutions of P by Nb can be estimated. It should also be noted that although Raman spectroscopy shows significant bands associated with Nb, there is only up to 2.7 mol% Nb_2O_5 in the glass network, while B_2O_3 is present at approximately 60% (Table 1), that is, the glass network becomes less connected.

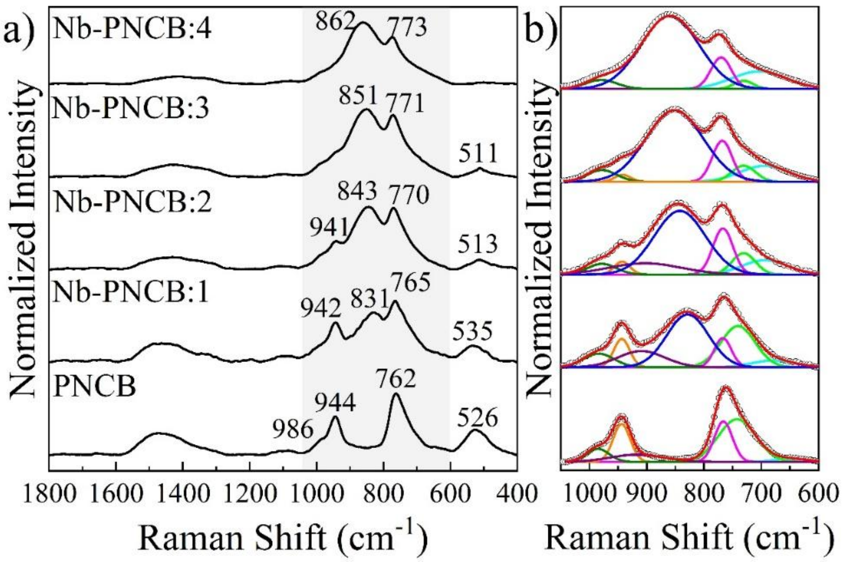

Figure S4: Molecular characterization of Nb-incorporated borate glasses. a) Raman spectra and b) Gaussian deconvolution of the Raman spectra to determine the structural changes caused by Nb addition.

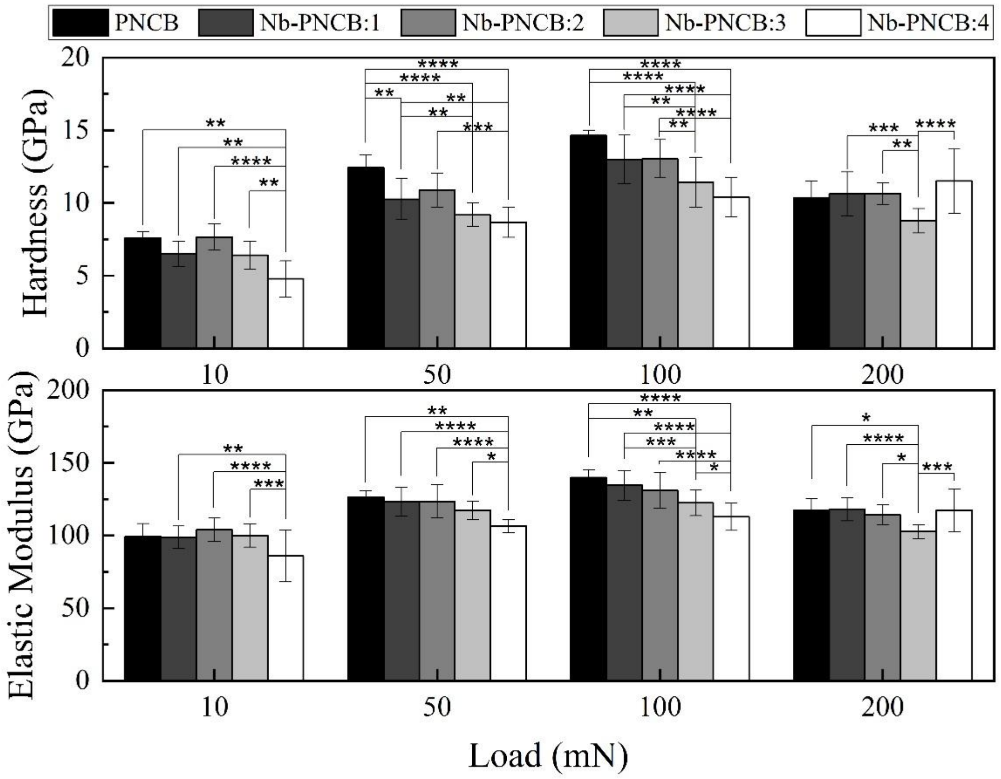

Figure S5: Hardness and elastic modulus of Nb-incorporated borate glasses generated by nanoindentation equipped with a Berkovich indenter and obtained in different loads. Statistical analysis was performed using two-way ANOVA followed by Tukey's post-hoc test. Significant differences are indicated as follows: *p < 0.05, **p < 0.01, ***p < 0.001, and ****p < 0.0001.

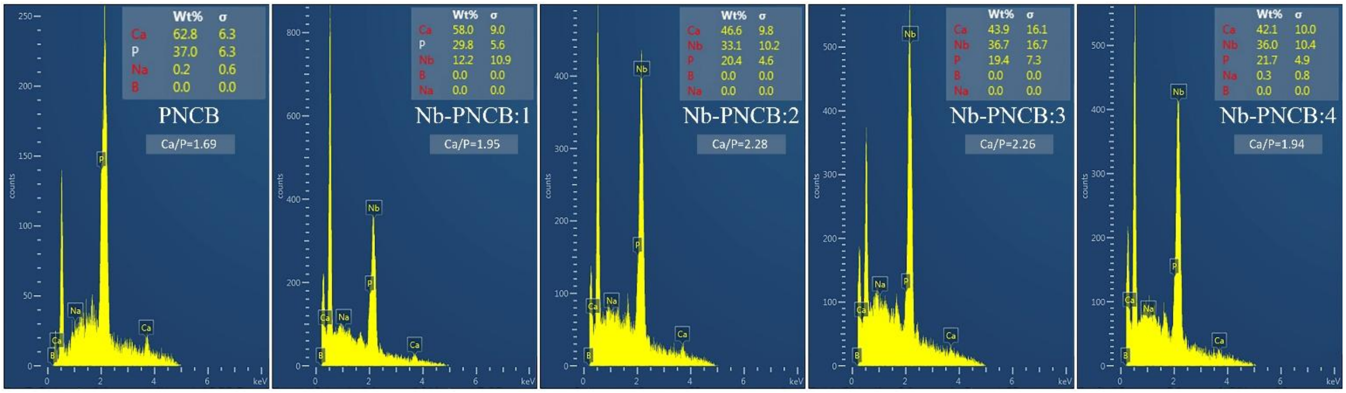

Figure S6: Energy-dispersive X-ray spectroscopy (EDS) spectra of Nb-incorporated borate glasses. The spectra show the elemental peaks and the corresponding weight percentages (wt.%) of each element. The Ca/P ratios are indicated for each sample.

REFERENCES:

(1) Meera, B. N.; Ramakrishna, J. Raman Spectral Studies of Borate Glasses. *Journal of Non-Crystalline Solids* **1993**, *159* (1–2), 1–21. https://doi.org/10.1016/0022-3093(93)91277-A.

(2) Konijnendijk, W. L.; Stevels, J. M. The Structure of Borate Glasses Studied by Raman Scattering. *Journal of Non-Crystalline Solids* **1975**, *18* (3), 307–331. https://doi.org/10.1016/0022-3093(75)90137-4.

(3) Pascuta, P.; Lungu, R.; Ardelean, I. FTIR and Raman Spectroscopic Investigation of Some Strontium–Borate Glasses Doped with Iron Ions. *J Mater Sci: Mater Electron* **2010**, *21* (6), 548–553. https://doi.org/10.1007/s10854-009-9955-7.

(4) Yiannopoulos, Y. D.; Chryssikos, G. D.; Kamitsos, E. I. Structure and Properties of Alkaline Earth Borate Glasses. *Phys. Chem. Glasses* **2001**, *42* (3).

(5) Sudhakar, B. K.; Chand, N. R. K.; Prasanna, H. N. L.; Rao, G. S.; Rao, K. V.; Dhand, V. Vibrational Spectral Analysis of Structural Modifications of Cr2O3 Containing Oxyfluoroborate Glasses. *Journal of Non-Crystalline Solids* **2010**, *356* (43), 2211–2217. https://doi.org/10.1016/j.jnoncrysol.2010.08.034.

(6) Yadav, A. K.; Singh, P. A Review of the Structures of Oxide Glasses by Raman Spectroscopy. *RSC Adv.* **2015**, *5* (83), 67583–67609. https://doi.org/10.1039/C5RA13043C.

(7) Koudelka, L.; Kalenda, P.; Mošner, P.; Montagne, L.; Revel, B. Structure–Property Relationships in Barium Borophosphate Glasses Modified with Niobium Oxide. *Journal of Non-Crystalline Solids* **2016**, *437*, 64–71. https://doi.org/10.1016/j.jnoncrysol.2016.01.017.

(8) Sene, F. F.; Martinelli, J. R.; L.Gomes. Synthesis and Characterization of Niobium Phosphate Glasses Containing Barium and Potassium. *Journal of Non-Crystalline Solids* **2004**, *348*, 30–37. https://doi.org/10.1016/j.jnoncrysol.2004.08.122.

(9) Cardinal, T.; Fargin, E.; Le Flem, G.; Couzi, M.; Canioni, L.; Segonds, P.; Sarger, L.; Ducasse, A.; Adamietz, F. Non Linear Optical Properties of Some Niobium (V) Oxide Glass. *Eur. J. Solid State Inorg. Chem.* **1996**, No. 33, 597–605.

(10) Kamitsos, E. I.; Chryssikos, G. D. Borate Glass Structure by Raman and Infrared Spectroscopies. *Journal of Molecular Structure* **1991**, *247*, 1–16. https://doi.org/10.1016/0022-2860(91)87058-P.

(11) Chatzipanagis, K. I.; Tagiara, N. S.; Kamitsos, E. I.; Barrow, N.; Slagle, I.; Wilson, R.; Greiner, T.; Jesuit, M.; Leonard, N.; Phillips, A.; Reynolds, B.; Royle, B.; Ameku, K.; Feller, S. Structure of Lead Borate Glasses by Raman, 11B MAS, and 207Pb NMR Spectroscopies. *Journal of Non-Crystalline Solids* **2022**, *589*, 121660. https://doi.org/10.1016/j.jnoncrysol.2022.121660.

(12) Kamitsos, E. I.; Karakassides, M. A.; Chryssikos, G. D. Vibrational Spectra of Magnesium-Sodium-Borate Glasses. 2. Raman and Mid-Infrared Investigation of the Network Structure. *J. Phys. Chem.* **1987**, *91* (5), 1073–1079. https://doi.org/10.1021/j100289a014.

(13) Meera, B. N.; Sood, A. K.; Chandrabhas, N.; Ramakrishna, J. Raman Study of Lead Borate Glasses. *Journal of Non-Crystalline Solids* **1990**, *126* (3), 224–230. https://doi.org/10.1016/0022-3093(90)90823-5.

(14) Yu, B.; Chen, B.; Yang, X.; Qiu, J.; Jiang, X.; Zhu, C.; Hirao, K. Study of Crystal Formation in Borate, Niobate, and Titanate Glasses Irradiated by Femtosecond Laser Pulses. *J. Opt. Soc. Am. B* **2004**, *21* (1), 83. https://doi.org/10.1364/JOSAB.21.000083.

(15) Iordanova, R.; Milanova, M.; Aleksandrov, L.; Shinozaki, K.; Komatsu, T. Structural Study of WO3-La2O3-B2O3-Nb2O5 Glasses. *Journal of Non-Crystalline Solids* **2020**, *543*, 120132. https://doi.org/10.1016/j.jnoncrysol.2020.120132.
